# MBenes: A Comprehensive Review of Synthesis Techniques and Energy Storage Capabilities

**DOI:** 10.1002/gch2.202500245

**Published:** 2025-08-26

**Authors:** MD. Tanvir Amin, Md. Shaib Hossain, Md. Muktadir Billah, Md. Arafat Rahman

**Affiliations:** ^1^ Department of Mechanical Engineering Chittagong University of Engineering and Technology Chittagong 4349 Bangladesh; ^2^ Department of Materials and Metallurgical Engineering Bangladesh University of Engineering and Technology Dhaka 1000 Bangladesh

**Keywords:** chemical exfoliation, hydrothermal, Lithium‐ion batteries, MBenes, optical, synthesis techniques, transition metal

## Abstract

This review thoroughly examines the potential of 2D transition metal borides (MBenes) for sophisticated energy storage applications. The investigation explores their structural stability, electrochemical characteristics, and a range of synthesis methods, including chemical and hydrothermal exfoliation procedures. Because of their strong mechanical characteristics, low diffusion energy barriers, and high theoretical capacities, MBenes perform better as anode materials for lithium‐ion batteries (LIBs) than other 2D materials like MXenes. Functionalized MBenes also show adaptability in various applications, such as lithium–sulfur batteries and nitrogen reduction catalysis. Their actual application is hampered by issues including oxidation and surface imperfections, despite their encouraging qualities. This analysis demonstrates MBenes as a promising anode material for next‐generation energy storage devices and highlights the necessity for more research into scalable, eco‐friendly synthesis techniques for achieving sustainability.

## Graphical abstract

This manuscript comprehensively reviewed 2D metal borides (MBenes) for advanced energy storage and explored synthesis techniques impacting stability and efficiency.

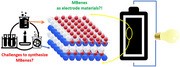

## 1 Introduction

Greater expertise in energy transformation and storage is required as human society gradually moves toward a greater reliance on knowledge and intelligence. Energy storage systems must be developed since renewable energy sources like wind, geothermal, solar, and tidal energies are inherently unpredictable and irregular.^[^ ^1^, ^2^, ^3^ ^]^ Because of its superior energy density, excellent cycle efficiency, and wide range of applications, the storage of electrochemical energy stands out among other energy storage methods.^[^ ^4^, ^5^ ^]^ However, locating solid and efficient electrodes is a common issue with many systems. This difficulty has impeded the development of related technologies and had a major impact on the field of electrolytic energy storage.^[^ ^6^ ^]^ Even while rechargeable batteries have a high energy density, their low power density limits their use in heavy power‐demanding applications.^[^ ^7^ ^]^

**Figure 1:**
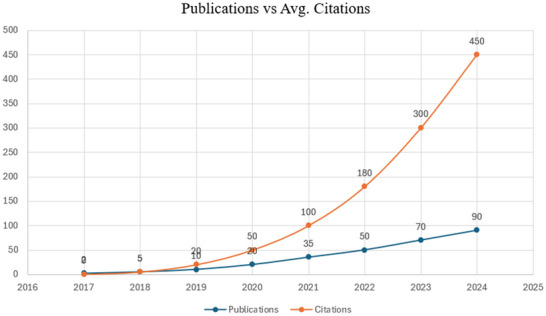
An overview of research contributions in the field of MBenes reveals a steady rise in the number of publications from 2017 to the present, reflecting the growing scientific interest in this area. (Data Source: Clarivate Web of Science).

2D materials, which are the focus of the forthcoming generation of electrode materials, are highly favored for a variety of applications because of their special qualities, which include a large surface area, atomic‐scale thickness, activated responsiveness, improved electron‐hole mobility, and great mechanical robustness.^[^ ^8^ ^]^ 2D transition metal borides, or MBenes, have emerged because of recent developments in material science and hold promise for enhancing energy storage systems. MBenes perform better electrochemically than MXenes due to their higher surface activation energy, which improves electrolyte‐ion interaction, and lower bond energy, which makes etching easier.^[^ ^9^ ^]^ These characteristics result in enhanced conductivity and more effective energy storage.^[^ ^1^ ^]^ Exploration of materials such as graphene,^[^ ^10^ ^]^ transition metal disulfides,^[^ ^11^ ^]^ hexagonal nitride of boron,^[^ ^12^ ^]^ and MXenes (evolution metal carbide compounds and nitrides) has advanced significantly in the research of 2D materials.^[^ ^13^, ^14^ ^]^ Among these, MBenes have drawn special interest due to their remarkable electrical conductivity, remarkable thermal conductivity, and remarkable anti‐oxidation qualities.^[^ ^15^, ^16^ ^]^ It is noted that studies have shown that MBenes have a very high modulus of Young and remarkable stability due to their isotropic structure.^[^ ^17^ ^]^ The MAB phases, which are the predecessors of MBenes, provide insight into their mechanical characteristics and crystal structures. MXenes, or transition metals such as carbides, nitric oxide, and carbonitrides, have previously demonstrated promise in enhancing lithium‐ion batteries, supercapacitors, water splitting for hydrogen production, and chemical reactions as electrocatalysts.^[^ ^16^ ^]^ M_n + 1_X_n_, where n is an integer between 1 and 3, X is either C or N, and M is an early transition metal, is the hexagonal close‐packed (HCP) structure that defines MXenes.^[^ ^18^ ^]^ It is worth mentioning that their different applications have been recognized and are being predicted both theoretically and experimentally which as shown in **Figure** **1**.

**Figure 2:**
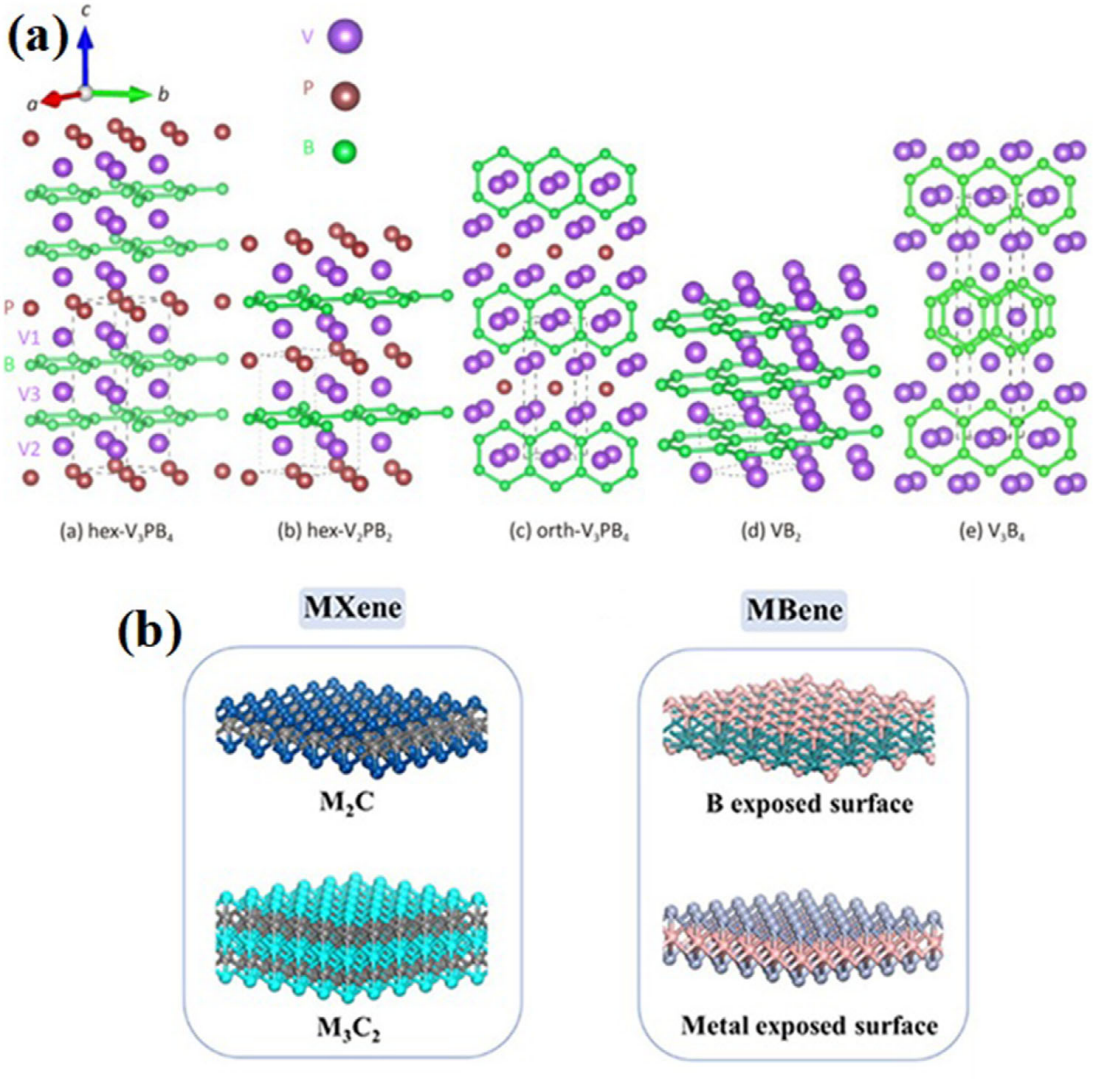
a) Different MAB phase crystal shapes (Adapted with permission)^[^ ^21^ ^]^ and b) MXenes and MBenes' structures. (Adapted with permission).^[^ ^20^ ^]^

The MAB phases, which are the predecessors of MBenes, provide insight into their mechanical characteristics and crystal structures. MXenes, or transition metal carbides, nitrides, and carbonitrides, have previously demonstrated promise in enhancing lithium‐ion batteries, supercapacitors, water splitting for hydrogen production, and chemical reactions as electrocatalysts.^[^ ^16^, ^19^ ^]^ M_n + 1_X_n_, where n is an integer between 1 and 3, X is either C or N, and M is an early transition metal, is the hexagonal close‐packed (HCP) structure that defines MXenes.^[^ ^18^ ^]^ Boride‐based structures, more especially ternary transition‐metal borides, or MAB phases have been included in this structural framework. Chemical exfoliation, which includes removing the A layer (mostly In or Al) to generate 2D materials, is how MBenes are created from MAB phases. For example, bulk MAB phases such as Ti_2_InB_2_, MoAlB, and Cr_2_AlB_2_ have been used as precursors to successfully synthesize MBenes like TiB, MoB, and CrB, respectively.^[^ ^20^ ^]^ Depending on how strong the bonds are during the etch of MAB phases, various forms of 2D MBenes, such as MB, M_2_B_3_, and M_3_B_4_, arise. **Figure** **2**a shows the crystalline structures of a number of MAB phases, demonstrating how their distinct characteristics aid in the formation of MBenes.^[^ ^21^ ^]^ Since both MBenes and MXenes include exfoliating the A layer of their layered precursor structures, there are similarities between their manufacturing techniques. The resultant MBenes have special electrical and mechanical properties that increase their potential for a range of uses. The structural similarities among MBenes and MXenes are shown in Figure 2b, which also emphasizes how respective layered structures provide the basis of their characteristics.^[^ ^22^ ^]^ Therefore, it was suggested by Khazaei and colleagues that orthorhombic 2D MBenes (MB, M_2_B_3_, and M_3_B_4_) with rectangular lattice structures can change into single or many layers of boron sheets that resemble graphene sandwiched between transition metals.^[^ ^23^ ^]^

Modern energy infrastructure increasingly relies on advanced electrochemical storage systems, with lithium‐ion batteries and supercapacitors serving as foundational technologies for applications spanning portable electronics to renewable energy integration.^[^ ^24^ ^]^ LIBs have attracted particular interest for grid‐scale deployment due to their superior energy density and cycling stability,^[^ ^25^, ^26^ ^]^ yet fundamental constraints, including theoretical energy limits (≈300–350 Wh/kg),^[^ ^27^ ^]^ safety risks, and resource scarcity, continue to challenge their universal adoption.^[^ ^28^ ^]^

Complementary to batteries, supercapacitors offer distinct advantages through their exceptional power delivery and cycle durability,^[^ ^29^ ^]^ though their comparatively modest energy density restricts standalone applications. This technological landscape has driven intensive research into novel electrode materials capable of bridging the performance gap between these systems,^[^ ^30^, ^31^, ^32^ ^]^ with 2D nanomaterials emerging as particularly promising candidates. Among these, transition metal borides (MBenes) represent a rapidly developing material class demonstrating remarkable electrochemical properties.

Unlike many nanomaterials requiring extreme synthesis conditions, MBenes can be produced through relatively mild fabrication processes, facilitating potential industrial scalability. Multiple MBene variants, including titanium, magnesium, molybdenum, chromium, and zirconium borides have exhibited exceptional charge storage characteristics. Theoretical modeling suggests yttrium diboride (Y_2_B_2_) could achieve remarkable specific capacities exceeding 800 and 400 mAhg^−1^ for lithium‐ion and sodium‐ion batteries, respectively. Experimental work on molybdenum and iron borides has confirmed their fast ion transport kinetics and structural stability during cycling, while surface‐modified variants show particular promise for improving sulfur utilization in next‐generation battery chemistries.^[^ ^33^ ^]^ Notably absent from current MBene research is a systematic investigation of their applicability for zinc‐ion storage, despite ZIBs' growing recognition as sustainable alternatives to lithium‐based systems. The development of MBene electrodes tailored for zinc electrochemistry could unlock new possibilities for safe, cost‐effective energy storage, representing an important frontier in materials science research.

This review has provided a comprehensive overview of the synthesis techniques, properties, and applications of MBenes, emphasizing the various methods such as chemical reduction, chemical exfoliation, mechanical exfoliation, and the hydrothermal method, alongside other approaches. The excellent properties of MBenes make them promising candidates for a variety of energy storage applications such as LIBs. This paper presents an analytical viewpoint on the performance metrics of MBene as an anode material of LIBs. However, challenges remain, particularly regarding their stability, which must be addressed for practical, large‐scale applications in energy storage and beyond. Recent research advancements and the necessity of further research to overcome these hurdles and to optimize the performance of MBenes have also been depicted in this paper.

## 2 Types of MBenes

Important discoveries on the magnetic properties and structures of MoAlB and Fe_2_AlB_2_ were made by Stadelmaier and Jeitschko in the 1960s, which greatly accelerated the study of transition metal borides (TMBs). When Chaban and Kuz'ma discovered Cr_2_AlB_2_ in 1973, they increased the M_2_AB_2_ family's variety.^[^ ^22^ ^]^ They compared the ternary carbide and nitride structures of the M_n + 1_AX_n_ (MAX) phase with the (CrB_2_)_n_CrAl (*n* = 1–3) structure present in these TMB phases, taking inspiration from MXenes. They came up with the moniker “MAB phase” after realizing the structural similarities.^[^ ^18^ ^]^ As precursors to produce 2D transition metal borides (MBenes), MAB phases, which are composed of layered ternary transitional metal borides having orthorhombic structures, are crucial. Alternating stacks of M_2_B_2_ and Al layers characterize these phases. Six orthorhombic MAB phase types: M_2_AB_2_, M_2_A_2_B_2_, M_3_A_2_B_2_, M_4_AB_4_, M_3_AB_4_, and M_4_AB_6_ are defined by A or A_2_M layers and alternating transitional metallic boride layers.^[^ ^20^ ^]^

The synthesis of MBenes from MAB phases is similar of MXenes. The variations in bonding strengths between M─A and M─X bonds are taken advantage of by selective etching techniques. Mixed ionic and covalent M─X connections in MAX phases contribute to stability, whereas pure metallic M–A interactions make it easier to remove A layers. Similar to MAX phases, strong M─B bonds in MAB phases point to the possibility of forming novel 2D transition metal borides. Targeted etching to remove Al atomic layers and create MBenes is feasible because the bonding energy of M─B bonds in MAB phases is roughly double that of M─Al bonds.^[^ ^20^ ^]^ In terms of their magnetic characteristics, itinerant magnetism has been demonstrated for compounds containing Mn as the B element.^[^ ^34^, ^35^, ^36^, ^37^, ^38^, ^39^ ^]^ The possibility of creating MBenes is further demonstrated by simulations of HF incorporation via the M_2_AlB_2_ edge. This method has been used to synthesize Cr_2_B_2_, Mo_2_B_2_, W_2_B_2_, and Fe_2_B_2_, which inherit orthorhombic geometries from their parent MAB phases. For instance, by etching Cr_3_AlB_2_ and MoAlB using HCl and NaOH solutions at ambient temperatures, respectively, Zhang et al.^[^ ^40^ ^]^ and Almada et al.^[^ ^41^ ^]^successfully analyzed 2D Cr_2_B_2_ and Mo_2_B_2_.

**Figure 3:**
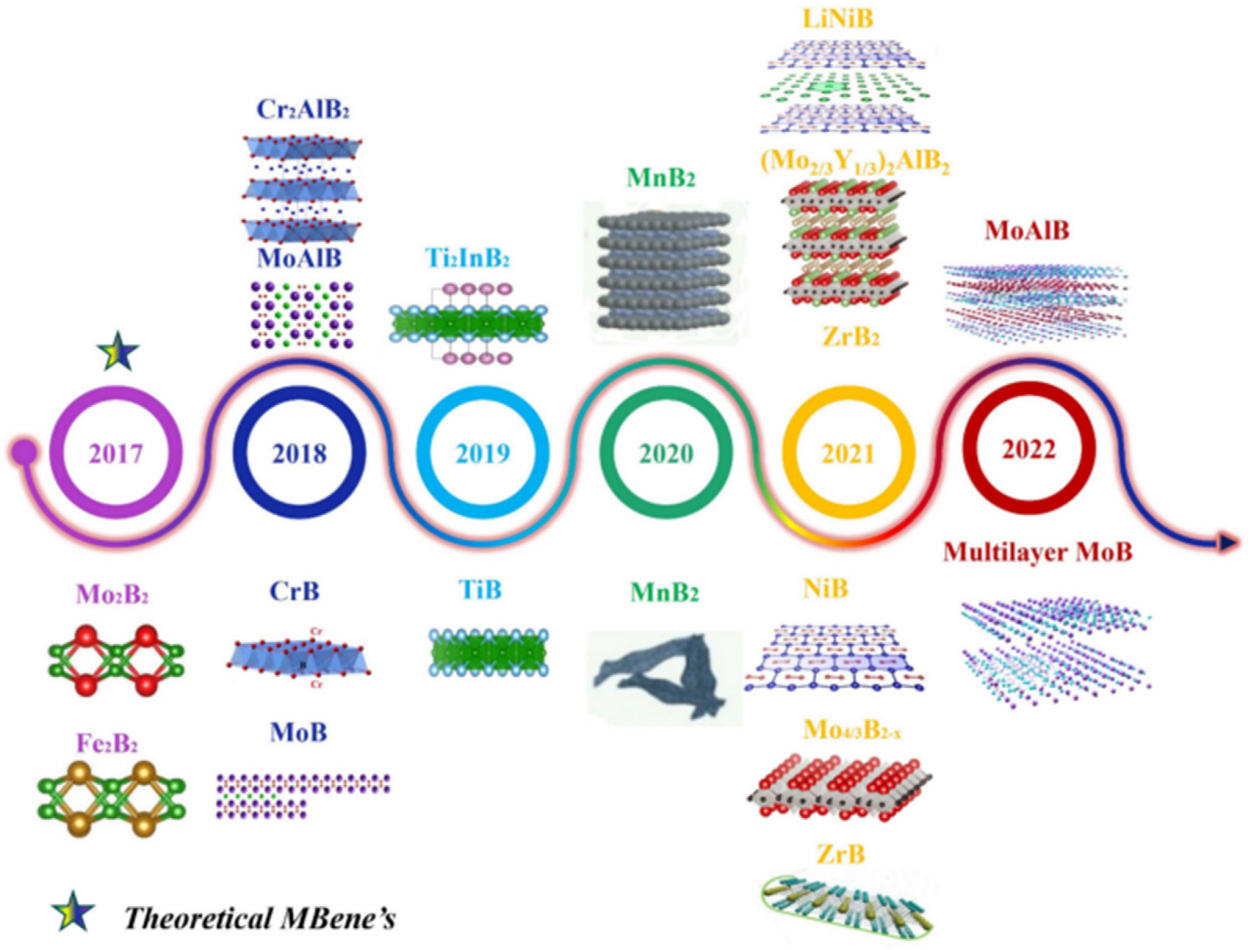
A timeline of MBenes significant discoveries across history (Adapted with permission).^[^ ^43^ ^]^

In contrast, MXenes' equivalents in hexagonal borides have been investigated. Hexagonal 2D M_2_B_2_ materials with structural similarities to M_2_X MXenes, including Ti_2_B_2_, Sc_2_B_2_, Cr_2_B_2_, V_2_B_2_, Zr_2_B_2_, Y_2_B_2_, and Mo_2_B_2_.However, it wasn't until Wang et al. efficiently synthesized hexagonal Ti_2_InB_2_ in 2019 that analogous hexagonal MAB phases were discovered, making it the only one produced to date.^[^ ^38^ ^]^ Significant turning points in the study and historical evolution of MBenes are highlighted,^[^ ^42^ ^]^ followed by extensive theoretical investigations of various MBene structures; this report further encompasses experimentally synthesized MBense in **Figure** **3**.^[^ ^43^ ^]^ Guo et al. utilized density functional theory (DFT) to show how MoB performs better electrochemically in LIBs.^[^ ^44^ ^]^ Alameda et al.^[^ ^41^ ^]^ demonstrated the Al deintercalation process from MoAlB using ADF‐STEM imaging. In addition, Bhaskari et al. demonstrated that Li could be stored between NiB layers and deintercalated from the multilayer polymorphs of LiNiB molecules.^[^ ^45^ ^]^ A promising option for sodium‐ion battery anodes, V_2_B_2_ was shown to be capable of strongly absorbing Na ions by Wei et al. In addition, Wei et al. documented the first application of MBenes in SCs, demonstrating enhanced electrochemical performance.^[^ ^46^, ^47^ ^]^ Based on crystal symmetries, the MBene family is divided into two groups: orthorhombic (orth‐MBenes) and hexagonal (hex‐MBenes). MBenes, which have the unifying chemical formula M_n_B_2n−2_, are derived from MAB phases and have alternating layers of transition metals and boron. Hex‐MBenes originate from hexagonal MAB precursors, whereas orthorhombic MAB precursors yield ortho‐MBenes.^[^ ^48^ ^]^ A sequence of hexagonal 2DM_2_B_2_ materials, such as Ti_2_B_2_, Sc_2_B_2_, Cr_2_B_2_, V_2_B_2_, Zr_2_B_2_, Y_2_B_2_, and Mo_2_B_2_, were documented by this same research group. These materials exhibited structural similarities to M_2_X MXenes. Similar hexagonal MAB phases, however, were not found. It is noted that Wang et. al. successfully synthesized hexagonal Ti_2_InB_2_, which is still the only hexagonal MAB phase that has been synthesized to date to our best knowledge.^[^ ^49^ ^]^

**Figure 4:**
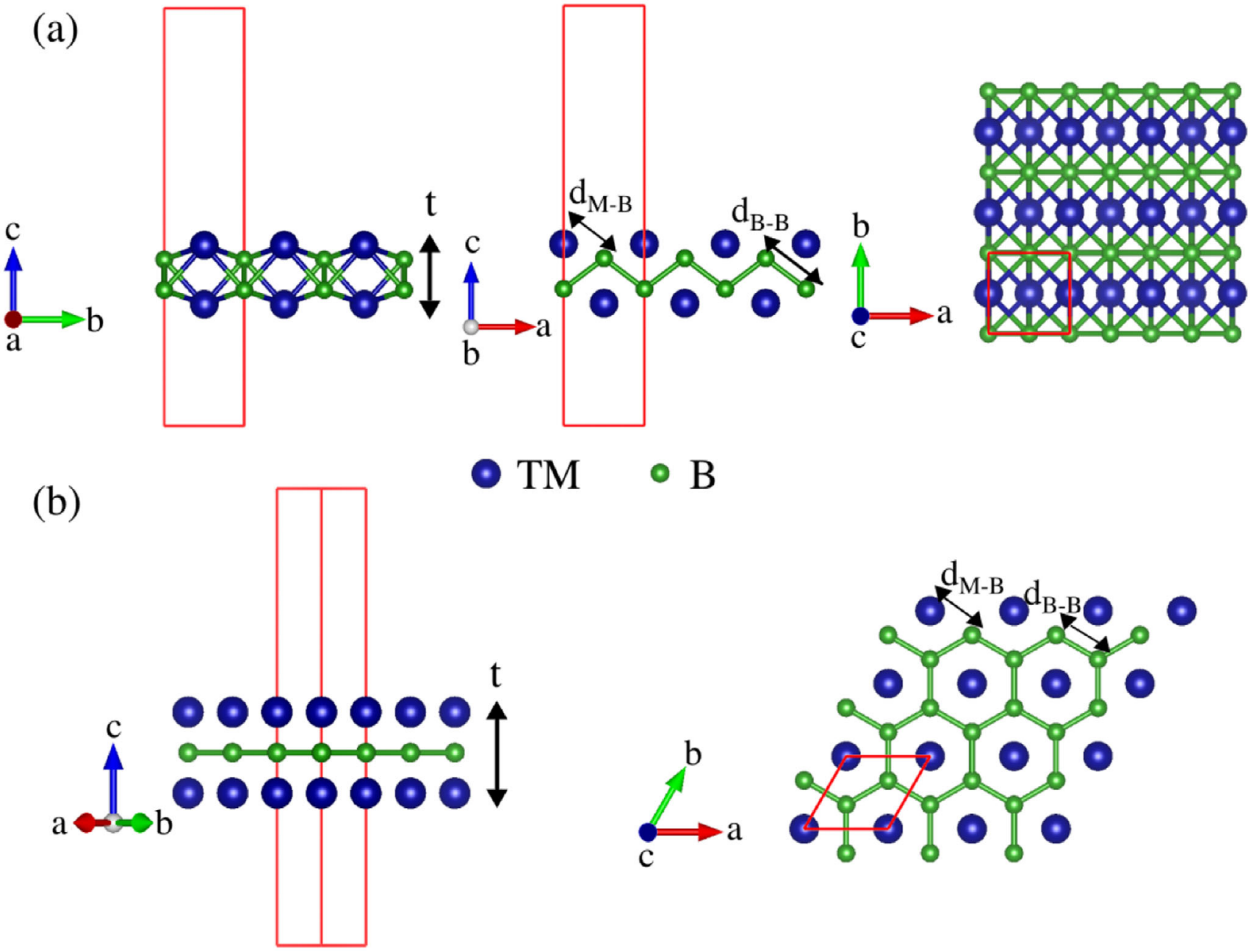
The reported atomic frameworks of a) ortho‐MBene and b) hex‐MBene structures of M_2_B_2_. (Adapted with permission).^[^ ^57^ ^]^

**Figure** **4** shows all known MBenes and their corresponding structures. It is noted that some borides, such as 2D Ti_2_B,^[^ ^50^ ^]^ Mo_2_B,^[^ ^51^ ^]^ and Hf_2_B^[^ ^52^ ^]^ are categorized as boron‐containing MXenes, even though conventional M_2_X MXenes and MBenes have similar geometries and chemical formulas. In contrast, 2D LIB^[^ ^45^ ^]^ and SIB^[^ ^53^ ^]^ are not considered MBenes since they do not have layered structures with alternating boron and transition metal layers. This category does not include other borides, such as MgB_2_, FeB_2_, TiB_2_, and tetragonal Mn_2_B_2_, which likewise differ structurally and compositionally from MAB‐derived MBenes.^[^ ^54^, ^55^, ^56^, ^57^ ^]^ Hexagonal MAB phases, such as (M_2/3_M _1/3_)_2_AlB_2_, serve as precursors to certain MBenes, showing structural similarities to hexa‐MBenes. These compounds feature unique in‐plane ordering of metal vacancies or elements. While borides such as MB_6_, M_5_B_2_, and M_10_B_4_ differ significantly from MAB‐derived MBenes, they highlight the diversity of transition metal borides.^[^ ^58^, ^59^ ^]^ The previous definitions and features of MBenes can be utilized to differentiate them from other 2D borides. First off, since they share analogous geometries and chemical formulas in common with traditional M_2_X MXenes, 2D Ti_2_B,^[^ ^55^ ^]^ Mo_2_B,^[^ ^56^ ^]^ and Hf_2_B^[^ ^51^ ^]^ should be categorized as new boron‐containing MXenes.

Furthermore, 2D LiB^[^ ^60^ ^]^ and NiB^[^ ^58^ ^]^ lack the layered structures that are made up of B layers and alternating transition metals. In addition, no MAB precursors have been identified in 2D MgB_2_,^[^ ^59^ ^]^ FeB_2_,^[^ ^61^ ^]^ TiB_2_,^[^ ^61^ ^]^ or tetragonal Mn_2_B_2_.^[^ ^62^ ^]^ Other transition‐metal borides have unique compositions and structures that differ significantly from those produced from the MAB phases. Samples of these were MB_6_ and M_5_B_2_ M_10_B_4_.^[^ ^63^, ^64^ ^]^ Therefore, they were not included in the MBene category.

## 3 Synthesis Approaches of MBenes

### 3.1 Chemical Reduction

The chemical reduction of metal salts using strong borohydrides is one of the most straightforward and effective techniques for synthesizing MBenes (2D metal borides) with tailored properties.^[^ ^22^, ^65^ ^]^ This method, which operates in liquid‐phase, is scalable and has proven successful in creating 2D transition metal boride (TMB)‐based nanostructures for applications in electrocatalysis and energy storage.^[^ ^65^, ^66^ ^]^ During the process, metal precursors like Co_2_B or Fe_2_B are mixed with boron sources and reduced using participants such as lithium or sodium borohydride under controlled conditions. Liu D et al. utilized this liquid‐phase reduction to generate 2D metal boride nanostructures for electrolytic purposes. The reaction is spontaneous and releases heat, typically completing in just a few minutes. It results in a black residue, which can be separated and dried.^[^ ^66^ ^]^ The borohydride acts both as a reducing agent and a source of boron. Initially, the synthesized TMB nanosheets are amorphous but crystallize when heated at high temperatures.^[^ ^66^ ^]^ This process provides fine control over the MBenes' composition, structure, and morphology, making them highly effective for electrocatalytic outcomes such as oxygen evolution (OER) and hydrogen evolution (HER).^[^ ^66^, ^67^ ^]^

### 3.2 Chemical Exfoliation

Liquid‐phase exfoliation is a common method used to break down stacked metal borides into 2D structures, particularly effective for MB_2_‐type metal borides that have an AlB_2_ structure. In recent years, this technique has been applied to various AlB_2_‐type borides, such as MgB_2_, AlB_2_, TiB_2_, ZrB_2_, HfB_2_, NbB_2_, TaB_2_, CrB_2_, and MnB_2_, successfully producing nanosheets of 2D metal borides.^[^ ^68^, ^69^ ^]^ By adjusting the recrystallization period during synthesis, it is possible to obtain different MBene morphologies, consisting of nanograins, nanodots, nanosheets, nanoflakes, and nanogarlands.^[^ ^70^ ^]^ The efficiency and quality of MBenes obtained through exfoliation depend significantly on the choice of solvent, and recent research is focused on developing new solvent strategies to achieve higher yields and better‐quality materials.^[^ ^69^ ^]^ One promising approach is microwave‐assisted hydrothermal etching, which converts MoAlB into 2D MoB MBenes with a distinctive accordion‐like structure. This unique structure enhances its electrochemical outcomes in sodium‐ion batteries, delivering a reversible ability of 196.5 mAhg^−1^.^[^ ^71^ ^]^ In addition, advancements in chemical exfoliation have enabled the synthesis of single‐layer MBenes, overcoming challenges posed by orthorhombic structures.^[^ ^72^ ^]^ These methods are environmentally friendly and scalable, especially in the case of electrochemical exfoliation, making them viable for large‐scale production.^[^ ^73^ ^]^ However, there is still a need to further refine the process to improve yield, quality, and structural integrity.^[^ ^74^ ^]^

### 3.3 Mechanical Exfoliation

Metal‐boride‐derived 2D nanostructures can be effectively synthesized using solid‐state mechanical exfoliation, a method known for its high yield.^[^ ^75^, ^76^ ^]^ Although metal borides like TiB_2_, NbB_2_, MgB_2_, Ni_2_B, and VB_2_ have primarily been produced submicrometer particles through ball milling,^[^ ^75^ ^]^ recent findings suggest that ball milling and mechanical grinding can successfully exfoliate metal borides into 2D nanoforms.^[^ ^77^ ^]^ The yield of pristine MBenes can be significantly improved by employing suitable high‐energy ball milling techniques with optimal parameters, such as ball‐powder ratio and milling duration. Furthermore, the straightforward nature of the synthesis equipment allows for scalability in commercial applications, making it essential to investigate the potential uses of MBenes produced through ball milling or mechanical grinding in fields like electronics, catalysis, and energy storage.^[^ ^77^ ^]^

The mechanical exfoliation of 2D MBenes involves separating thin layers or flakes from their bulk forms, resulting in a high yield of material. This approach, commonly known as the “Scotch tape method,” involves manually peeling layers from bulk materials to create mono‐ or few‐layered MBenes.^[^ ^78^ ^]^ The effectiveness of mechanical exfoliation can be enhanced by optimizing parameters such as pressure, temperature, and adhesive materials, which aid in producing MBenes. For instance, Jia et al.^[^ ^79^ ^]^ displayed a mechanical peeling technique to obtain 2D structures, and an extensive understanding of the microscopic mechanisms involved in peeling from M_2_AB_2_ to M_2_B_2_ supports the practicality of this method. Data illustrating the peeling process and stress–strain characteristics indicate that the strength of the B─Mn bonds around Al atoms remains relatively constant as tensile strain raises from 1% to 30%.

In summary, the mechanical exfoliation method for synthesizing MBenes has gained considerable curiosity due to its potential in several applications, particularly in energy storage. This technique effectively separates layers from bulk materials, yielding high‐quality nanosheets. Solid‐state mechanical exfoliation uses mechanical forces for this purpose, while micromechanical exfoliation employs adhesive materials to enhance the quality and quantity of exfoliated MBenes.^[^ ^80^, ^81^ ^]^

The production of MBene nanosheets through mechanical exfoliation comprises three distinct phases: initial preparation, the exfoliation process itself, and subsequent refinement steps. In the preparatory stage, researchers typically fracture or pulverize the parent MBene crystals to create fresh surfaces, while simultaneously preparing ultra‐clean substrates to maximize material adhesion.^[^ ^82^, ^83^, ^84^ ^]^ For the actual exfoliation, several advanced methodologies have emerged: the conventional Scotch tape approach (accessible but inefficient), shear‐force techniques employing precision three‐roll mills, robotic platforms utilizing machine learning for process optimization, and environmentally benign supercritical CO_2_ methods that eliminate organic solvents.^[^ ^84^, ^85^, ^86^ ^]^ The final processing stage includes thorough solvent rinsing (typically using alcohols), centrifugal separation to purify the nanosheets, and precise deposition onto desired surfaces.^[^ ^83^, ^85^ ^]^ Key considerations influencing outcomes include the crystalline quality of source materials, careful calibration of operational parameters (including peeling dynamics and applied forces), and the ongoing challenge of industrial‐scale production – where continuous roll‐based systems and advanced milling technologies show particular promise.^[^ ^84^, ^87^ ^]^

Mechanical exfoliation offers several advantages, such as producing fewer defects compared to chemical methods, resulting in superior electrical and mechanical properties,^[^ ^81^ ^]^ as well as its adaptability for large‐scale production, which makes it suitable for industrial applications.^[^ ^88^ ^]^ Despite these advantages, alternative techniques like electrochemical exfoliation are being explored for their efficiency and scalability, showcasing a variety of synthesis methods available for MBenes.^[^ ^89^ ^]^

### 3.4 Hydrothermal Method

The hydrothermal and solvothermal methods are effective techniques for synthesizing MBenes, which are 2D carbides or nitrides of transition metals. These methods enable the precise formation of layered structures, a key factor in the unique properties of MBenes. In hydrothermal synthesis, reactions between metal and boron precursors occur under high‐pressure and high‐temperature conditions in liquid solutions, while solvothermal synthesis uses organic solvents as the medium. These processes are advantageous due to their relatively low temperatures, ease of controlling particle shape and size, and the capacity to create materials with specific crystal frameworks. Different MBenes, such as transition‐metal borocarbides and diborides, have been successfully synthesized through these methods, showing significant potential for applications in energy storage and catalysis. For instance, coordination polymers like [Fe(phen)(µ6‐bta)_1/2_]_n_ were synthesized using hydrothermal techniques, demonstrating a 2D polymeric structure.^[^ ^90^ ^]^ The versatility of these techniques is further demonstrated by the synthesis of complex metal‐organic frameworks (MOFs) using multicarboxylate and heterocyclic aromatic ligands.^[^ ^91^ ^]^ Structural integrity and topological details of the synthesized materials can be confirmed using X‐ray diffraction, as evidenced by several new MOFs.^[^ ^92^, ^93^ ^]^ A study by Xiong et al. highlighted the successful fluorine‐free synthesis of 2D MBenes using hydrothermal methods to be utilized as anodes in lithium‐ion batteries (LIBs). The electrochemical performance was evaluated using techniques like cyclic voltammetry (CV), electrochemical impedance spectroscopy (EIS), and galvanostatic charge–discharge measurements, showing promise for LIB applications.^[^ ^69^ ^]^ Analysis indicated that the 2D MoB achieved a 73.2% impurity level, with its transformation confirmed by XRD and SEM, depicting an accordion‐like structure post‐etching.^[^ ^94^ ^]^ Additionally, high‐resolution transmission electron microscopy (HRTEM) revealed a reversible specific ability of 144.2 mAh g^−1^ after 1000 charge‐discharge cycles, outperforming many previously reported MXene anodes.^[^ ^94^ ^]^ Although hydrothermal and solvothermal methods produce MBenes with excellent characteristics for energy storage and catalysis, chemical vapor deposition is another technique that offers advantages in scalability and uniformity for large‐scale production.

### 3.5 Others

Numerous methods have been explored for synthesizing MBenes, with electroless deposition standing out due to its effective use of reduction techniques to create metal‐diboride coatings. Most modern electroless deposition methods use NaBH_4_ as the reducing agent to form 2D metal boride nanostructures, also known as MBenes.^[^ ^95^ ^]^ However, to develop ultrathin films, hybrid synthesis strategies must be further advanced. Key factors, such as precursor flow rates, temperature, reaction time, substrate type, and pressure, need optimization since they play crucial roles in determining the film thickness and overall quality of MBenes. Fine‐tuning these factors will enable the creation of high‐level, ultrathin films suitable for a variety of future applications.^[^ ^96^ ^]^ Beyond electroless deposition, alternative synthesis techniques have been studied, including chemical vapor deposition (CVD), plasma‐assisted methods, electrochemical deposition, and HF etching. Plasma‐assisted approaches use plasma releases to trigger reactions among boron precursors and metal, while CVD ensures regulated MBene expansion by presenting reactive gases, including boron and metal, onto a substrate. Electrochemical synthesis contains depositing metal onto boron‐based materials under specific electrochemical circumstances, providing another means of fabricating MBenes with customized properties.^[^ ^94^ ^]^ It is noted that Jothi et al. demonstrated the synthesis of nanocrystalline molybdenum boride (MoB_2_) using redox‐assisted solid‐state metathesis (SSM) at a comparatively low temperature of 650 °C over 24 h, a method that is environmentally friendly and operates under mild conditions.^[^ ^97^ ^]^ Researchers are also investigating the removal of aluminum (Al) atom layers in MBenes, given their structural similarities to MXenes. For example, Mo_4/3_B_2_ was produced by etching (Mo_2/3_Y_1/3_)_2_AlB_2_ with a 40% HF solution at 33–35 °C, removing both Y and Al atoms and resulting in 2D Mo_4/3_B_2_ nanosheets greater than 50 nm with boron vacancies and functional AO, AOH, and AF groups.^[^ ^98^ ^]^

A range of synthesis techniques, comprising chemical reduction, mechanical exfoliation, ball milling, chemical exfoliation, and hydrothermal/solvothermal processes, have been applied to tailor MBene characteristics. Each approach offers distinct benefits and drawbacks, with the method's selection depending on the desired properties and target applications. As research advances, the development of MBenes continues to grow rapidly, driven by the need for components with improved catalytic capabilities and energy storage.

Recent research on 2D MBenes has been driven by the pursuit of atomically thin, delaminated structures similar to MXenes. Two primary synthesis strategies have been employed, differing in both the starting materials and the type of etching agents used. One approach utilizes MAB phases treated with either acidic or basic solutions, while the other relies on solvothermal fragmentation of bulk powders to form specific nanostructures. In addition, dealloying techniques have also been explored for producing 2D MBenes. A summary of these newly developed 2D boride materials and their functional characteristics is presented in **Table** **1**.

**Table 1:** Overview of the latest experimental advancements in synthesizing 2D boride phases and their corresponding properties.

Chemical composition	Synthesis method	Synthesis effect	Surface chemistry	Phase composition	Key findings	Refs.
MoB/Mo_2_B_2_	NaOH Etching of MoAlB/Mo_2_AlB_2_ MAB phase with etching temperature optimization	Al was partially deintercalated with phase transformations of MoAlB/Mo_2_AlB2 into a stable nanosheet heterostructure containing 2D Mo_2_AlB_2_ layers and amorphous aluminum oxide layers. MoAlB was etched only near the surface.	The formation of the aluminum oxide surface coating	Depending on the stage of the synthesis/etching process, different contents of MoAlB/Mo_2_AlB_2_ MAB phases and amorphous aluminum oxide	This work aimed at studying multi‐step topochemical pathway for 2D materials synthesis	[99]
MoB (thickness of 1.0‐1.5 nm)	MoAlB etching with NaOH or LiF/HCl	Al was removed partially in a stepwise manner with growing stacking faults, forming etched cavities	Some residues from the etching process could be present	Mostly partially etched MoAlB MAB phase with possible MoB monolayers formed in etched cavities	This study aimed at synthesizing 2D MBene. MoB layer slowly dissolves in the presence of HF at room temperature, which makes LiF/HCl not suitable for the deintercalation of Al from the MoAlB MAB phase	[47]
MgB_2_ (lateral sizes of 30 nm)	Ultrasound‐assisted chemical etching with CH_3_COOH, H_2_O_2_, and PEG4000	Obtaining 2D nanosheets.	Boric acid ester borate (B─O─PEG) with HO─B on the surface of MgB_2_	Major MgB_2_ with minor MgB_4_	High acid‐responsiveness for decomposition into H_2_, after combining with polyvinylpyrrolidone (PVP) and doxorubicin (DOX) facile agent for hydrogen chemotherapy	[100]
CrB (thickness of several to tens of nanomete)	Etching out Al atomic layers from the Cr_2_AlB_2_ MAB phase using diluted HCl	Investigated MAB was successfully etched into single nanosheets with high purity	Functional groups rather not present due to the high purity of the product confirmed by EDS analysis	A high content of 2D CrB with Cr_2_B_2_ as a transition phase.	This work aimed at the verification of a precursor for the synthesis of 2D CrB.	[101]
TiB (thickness of 10–20 nm)	Dealloying strategy adopted to exfoliate In layers from Ti_2_InB_2_ MAB phase	In layers were gradually extracted with increasing heating temperature, layered boride as the product	Calculations describing surface functional groups (F, Cl, OH, and O) that attributes to metal‐to‐semimetal transition	TiB with almost no In residue.	Analysis of possible Li+ or Na+ ions intercalation between TiB sheets to make it a promising material for Li‐ or Na‐ion batteries, confirmed by calculations	[49]
ZrB (7.4 nm thickness, 150 nm lateral size)	Microwave‐assisted chemical etching of ZrB2 powder	Successful etching into single nanosheets, which maintained the crystal structure of the pristine ZrB_2_ starting powder	Formation of borate ester between nanosheets after modification with HA. This allowed to prevent ZrB nanosheets aggregation in comparison to nonmodified flakes	No significant amount of any additional functional groups	HA‐modified ZrB_2_ flakes exhibit broad UV‐NIR absorption, enabling effective NIR‐photothermal response. Upon NIR irradiation, photopyrolysis of borate ester triggers HA detachment and ZrB_2_ aggregation, enhancing intratumoral retention and accumulation.	[102]

## 4 Properties of MBenes

**Figure 5:**
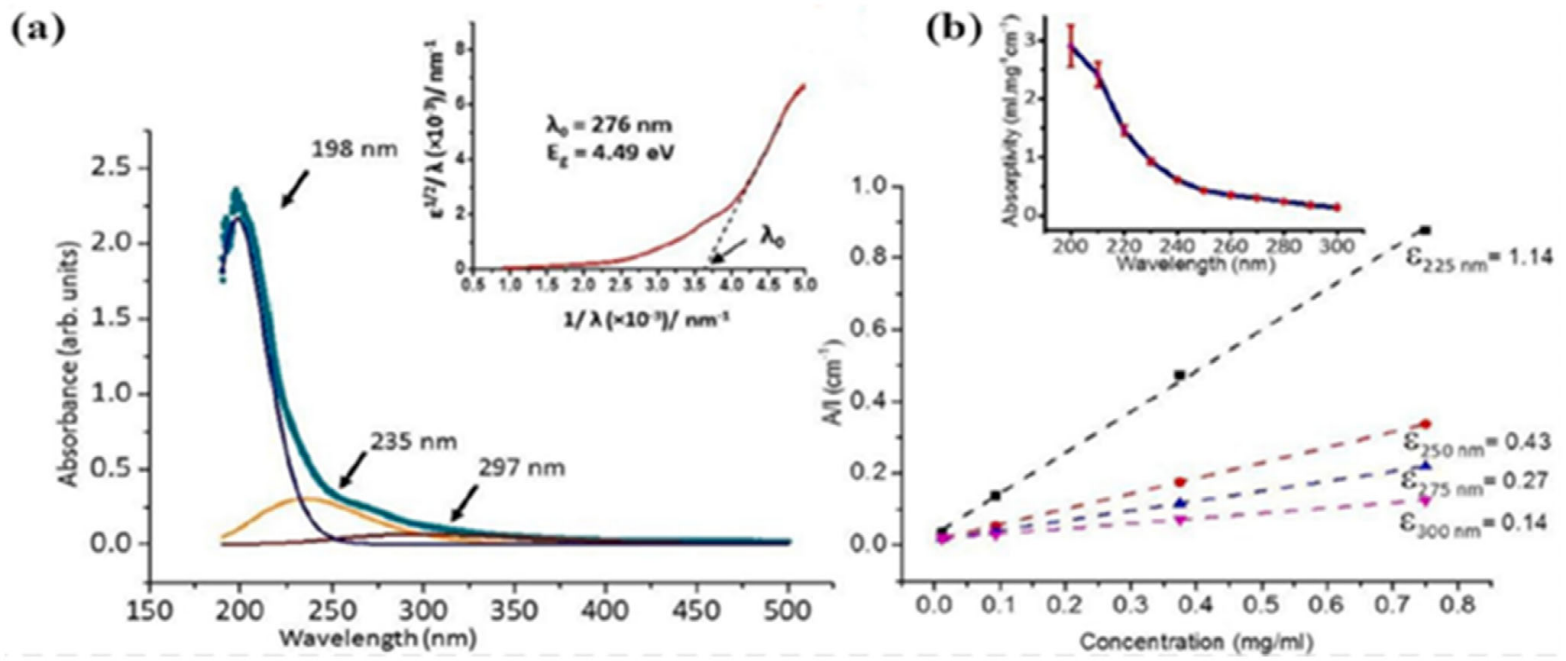
a) Absorbance spectrum of MgB_2_ nanosheets. b) Lambert‐Beer plot for the aqueous dispersion. (Adapted with permission).^[^ ^70^ ^]^

MBenes exhibit a range of characteristics that make them highly suitable for applications in energy storage and catalysis. Structurally, they consist of metal particles layered among boron sheets, creating a 2D structure that imparts significant stability and mechanical strength. The presence of metal particles allows for a diverse range of electronic properties and adjustable band structures, enabling tailored electronic gestures. Although research into the optical properties of MBenes is still developing, some notable studies, such as by Das et al.,^[^ ^70^ ^]^ have analyzed MgB_2_ nanosheets, determining a bandgap of about 4.49 eV (**Figure** **5**a,b). These materials show wide absorption and emission ranges, covering ultraviolet (UV) to near‐infrared (NIR) wavelengths, making them viable for optoelectronic devices. Specifically, their potential as absorbent materials in the UV and infrared regions has led to their use in coatings, nanostructures, and films for solar absorbers and UV filters. Additionally, through chemical modification and exfoliation, MBenes' bandgap can be tuned, expanding their application possibilities.^[^ ^22^ ^]^

The surface properties of MBenes can also be altered to improve their chemical reactivity, electrocatalytic efficiency, and mechanical strength. Functionalizing surfaces with elements like O, F, OH, or Cl alters their stability, conductivity, and catalytic activity. For example, Mo_2_B_2_ MBenes with functionalized surfaces are excellent for lithium–sulfur batteries due to their catalytic and anchoring properties.^[^ ^103^ ^]^ Khaledialidusti et al.^[^ ^104^ ^]^ reported that oxygen‐functionalized MBenes exhibit greater elastic constants than those functionalized with fluorine or hydroxyl groups, which implies increased mechanical stiffness. Surface terminations, such as OH, O, and F, significantly affect mechanical characteristics like Poisson's ratio and shear modulus, impacting overall mechanical behavior. While MBenes have a lower Young's modulus than MXenes, their phonon spectra lack imaginary modes, indicating strong kinetic stability at 300 K, as verified by MD models.^[^ ^105^ ^]^

**Figure 6:**
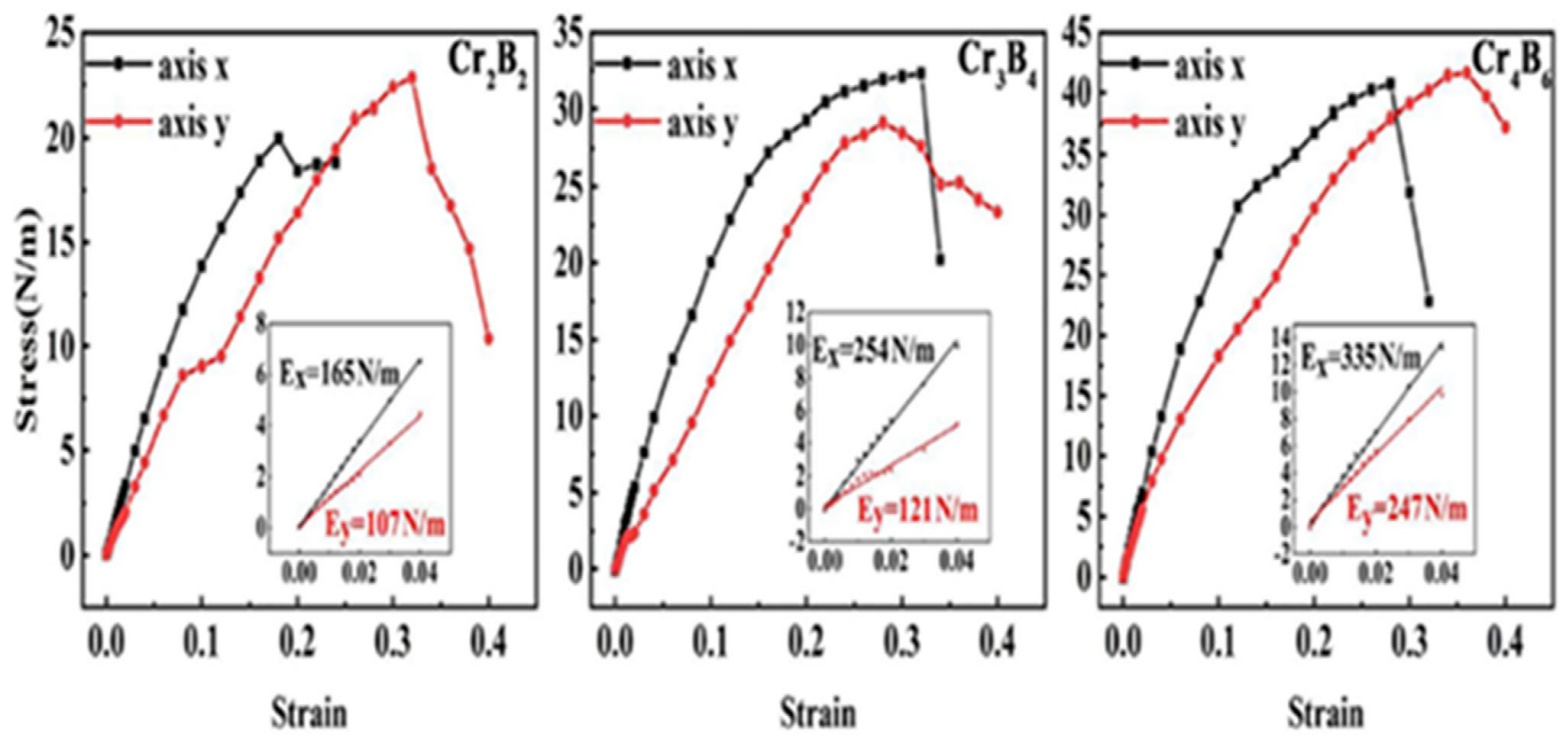
The stress–strain curve of Cr_n + 1_B_2n_ MBenes. (Adapted with permission).^[^ ^106^ ^]^

Regarding mechanical characteristics, the Young's modulus of Cr_4_B₆ has been calculated to be 335 N m^−1^ along the *x*‐axis and 247 N m^−1^ along the *y*‐axis (**Figure** **6**).^[^ ^106^ ^]^ Elastic constant calculations for Cr_n + 1_B_2n_ suggest they are mechanically steady according to the Born criteria. Guo et al.^[^ ^63^ ^]^ reported in‐plane stiffness values for several MBenes, with Au_2_B exhibiting the lowest stiffness (85 N m^−1^) and V_3_B_4_ the highest (395 N m^−1^). High in‐plane stiffness helps prevent layer distortion under gravity, enabling MBenes to create unsupported films. In addition, Khaledialidusti et al.^[^ ^104^ ^]^ demonstrated that surface functionalization significantly enhances mechanical characteristics like Young's modulus, elastic constants, and shear modulus. These surface groups also affect the electronic work function, which influences MBenes' overall stability.

MBenes also offers potential in electrocatalysis and spintronics. Xiaowei et al.^[^ ^107^ ^]^ examined ten 2D metal borides for nitrogen reduction reaction (NRR) catalysis, observing strong covalent bonds on the active surfaces. Research has further revealed magnetic properties in Cr‐, Mn‐, and Fe‐based MBenes, with Mn_2_B_2_ displaying ferromagnetic behavior above room temperature and Cr_2_B_2_ and Fe_2_B_2_ exhibiting both ferromagnetic and antiferromagnetic properties. These materials are promising for superior‐performance batteries and magnetic gadgets because of their ferromagnetic properties and high Curie temperatures.^[^ ^108^, ^109^, ^110^, ^111^ ^]^

In summary, MBenes display excellent mechanical stability, temperature resistance, and corrosion resistance, creating strong applicants for electronic, catalytic, and energy storage applications.

## 5 Applications of MBenes

Amongst the many benefits of MBenes' layered nanostructures are their excessive specific surface area, quick reaction kinetics, intensely active catalytic centers, and effective charge delivery. MBenes are seen as interesting alternatives for catalytic applications and energy storage due to these characteristics.^[^ ^112^ ^]^ Creating superior‐performance electrode contents for energy storage is a task that calls for constant advancements. The growth of superior‐performance lithium‐ion batteries is still ongoing, even after 30 years of research.^[^ ^113^ ^]^

**Figure 7:**
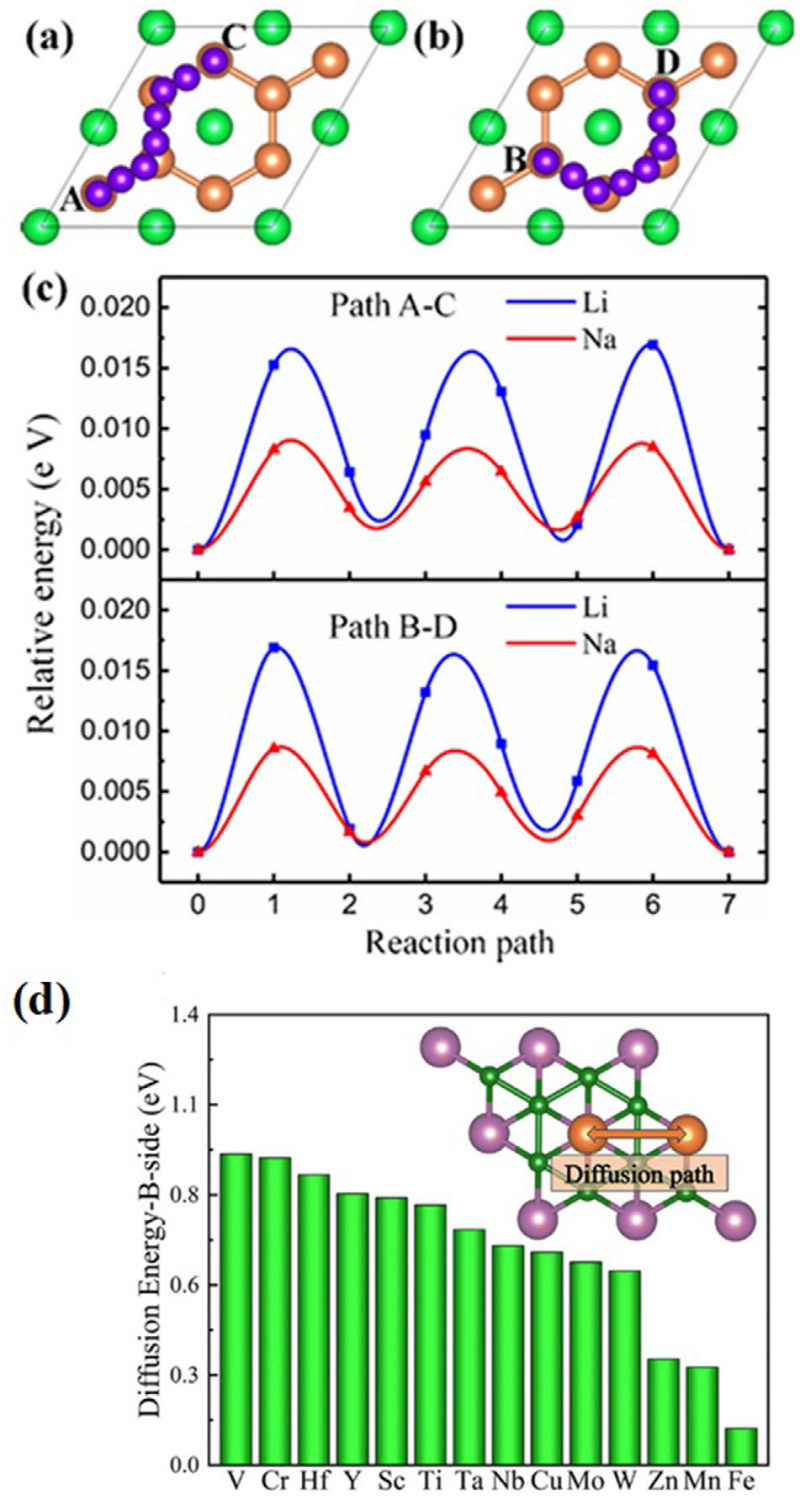
Li/Na Ion Diffusion Pathways and Energy Barriers in Ti_2_B_2_ MBene. a,b) Top views of diffusion paths A–C and B–D; Ti, B, and Li/Na atoms shown as green, orange, and violet spheres. c) Energy profiles of diffusion paths. (Adapted with permission),^[^ ^116^ ^]^ d) Li diffusion path and energy barrier on B‐side surface (Adapted with permission).^[^ ^120^ ^]^

Two‐dimensional materials behave differently from their bulk counterparts, giving rise to unique features such as larger surface area, improved ionic conductivity, many intercalation sites, and shorter diffusion lengths. Their flexibility in a range of applications is facilitated by these attributes.^[^ ^114^ ^]^ Anode battery materials are being developed by integrating effective redox chemistry with the special qualities of 2D materials. However, in the realm of energy storage, transition‐metal borides (MBenes) have not received as much focus as their predecessors, such as nitrides (metallic transition metal nitrides), carbides (MXenes), and phosphides (metallic transition metal phosphides). This opens up the possibility of investigating MBenes in energy‐related applications further.^[^ ^115^ ^]^ It is noted that Ti_2_B_2_ has a superior theoretical specific capability of 456/342 mAhg^−1^ and an ultralow diffusion energy barrier of 0.017/0.008 eV when utilized as an anode material for lithium/sodium ion batteries (**Figure** **7**a).^[^ ^116^, ^117^ ^]^ In addition, even after the absorption of three layers of metal ions, there is a very slight volume change.^[^ ^116^ ^]^ It is noted that Y_2_B_2_ has an excellent diffusion rate of 0.010/0.013 cm^2^s^−1^, a theoretical capacity of 806.31/403.16 mAhg^−1^, an initial open‐circuit voltage of 0.43/0.45 V for lithium/sodium ion batteries, and a barrier of diffusion of 0.013/0.008 eV (Figure 7b).^[^ ^118^ ^]^ Using V_2_B_2_ as an anode material, it is observed that the lowest barriers of diffusion energy of 0.22 and 0.13 eV for Li/Na‐ions, respectively (Figure 7c,d)). However, it can adsorb three layers of Li/Na atoms, with maximum theoretical capacities of 968 and 614 mAhg^−1^ for lithium/sodium ion batteries, respectively.^[^ ^119^ ^]^ Hence, high theoretical capacities and low diffusion energy barriers indicate that Ti_2_B_2_, V_2_B_2_, and Y_2_B_2_ are viable materials for the anode for lithium/sodium ion batteries.^[^ ^120^ ^]^ It is worth mentioning that the adsorption amount of metal atoms of V_2_B_2_ functionalized by oxygen atoms, for example, increases when the surface diffusion energy barrier decreases and increases, respectively.^[^ ^119^ ^]^ Functionalized Mo_2_B_2_O_2_ may also effectively inhibit the shuttle effect when used in lithium–sulfur batteries. In addition, it exhibits extremely low diffusion energy barriers and less decomposition energy barriers for Li_2_S.^[^ ^121^ ^]^

A summary of MBenes' performance in rechargeable batteries is shown in **Table** **2**. The theoretical specific capacity is significantly higher than that of certain other 2D materials, such as graphite and Ti_2_C_2_. It is worth mentioning that MBenes offer more possibilities in LIBs, SIBs, and MIBs due to their distinct structure from MXenes.^[^ ^122^ ^]^

**Table 2:** An overview of the primary functions of MBenes as anode materials for rechargeable batteries.

Materials	Methods	Type of battery	Diffusion energy [meV]	OCV [V]	Specific Capacity [mAh g^−1^]	Refs.
Ti_3_C_2_	Computational analysis	LIBs	280	0.62	320	[123]
Mo_2_B_2_	First‐principal estimates	LIBs	270	0.41	444	[13]
		MIBs	840	0.84	502.1	[124]
Graphite	DFT	LIBs	400	–	372	[125]
TiB	DFT	LIBs	20	0.33	480	[49]
		SIBs	20	0.17	480	[49]
Ti_2_B_2_	DFT	LIBs	17	0.526	456	[116]
		SIBs	8	0.502	342	[116]
TiB3	DFT	LIBs	38	0.156	1335.04	[126]
		SIBs	157	0.195	667.52	[126]
V_2_B_2_	First‐principal estimates	LIBs	220	–	969	[119]
		SIBs	130	–	614	[119]
	DFT	SIBS	11	0.65	814	[48]
V_2_B_2_O_2_	First‐principal estimates	LIBs	390	0.57	812	[119]
		SIBs	420	0.41	547	[119]
VB	computational assessment	LIBs	264	0.797	390.168	[127]
		SIBs	85	0.553	316.273	[127]
Cr_2_B_2_	DFT	LIBs	280	–	696	[119]
		SIBs	170	–	492	[119]
		MIBs	380	0.53	853.4	[124]
Mn_2_B_2_	First‐principal estimates	LIBs	290	–	679	[119]
		SIBs	170	–	483	[119]
Ti_2_B	DFT	MIBs	80	0.101	3018.41	[128]
		LIBs	90	0.532	503.07	[128]
		SIBs	110	.432	503.07	[128]
Tetr‐Mo_2_B_2_	Computational analysis	LIBs	29	0.835	251	[129]
		SIBs	10	0.515	251	[129]
Tri‐ Mo_2_B_2_	Computational analysis	LIBs	23	0.407	251	[129]
		SIBs	13	0.383	188	[129]
Fe_2_B_2_	DFT	LIBs	240	0.33	665	[13]
T‐Mo_2_B	First‐principal estimates	LIBs	37	0.628	264	[56]
H‐Mo_2_B	First‐principal estimates	LIBs	50	0.386	74.18	[56]

**Figure 8:**
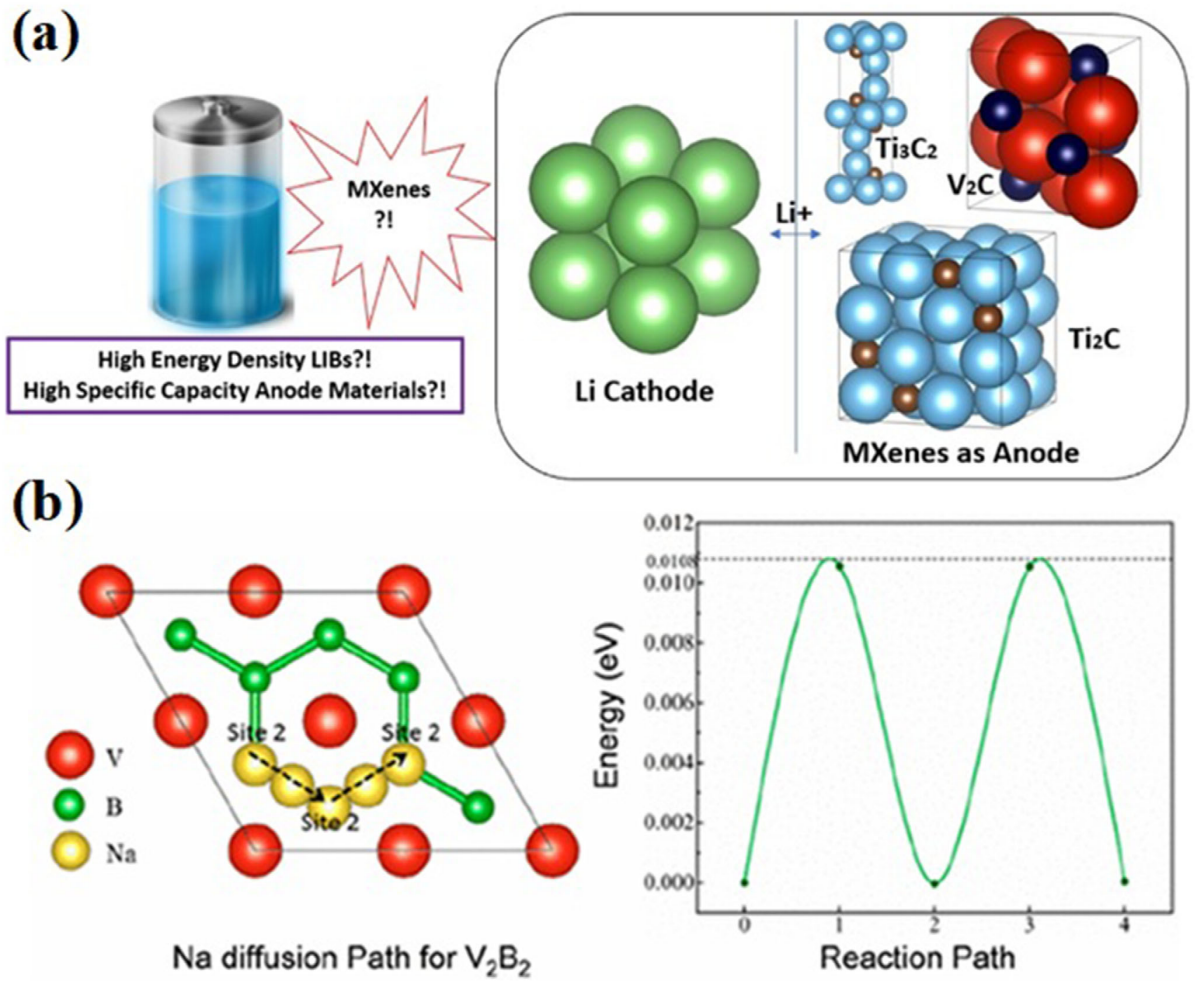
a) Anode materials based on MXene are shown schematically for LIBs (Adapted with permission),^[^ ^9^ ^]^ b) Top view of the Na diffusion path for V_2_B_2,_ indicated by the black dotted arrows and the corresponding diffusion barriers for Na (green solid line) on V_2_B_2_. (Adapted with permission).^[^ ^48^ ^]^

Due to their long cycle life, high‐power density, energy efficiency, and large capacity, lithium‐ion batteries are extensively used. In **Figure** **8**a, the construction and operation of the LIBs are depicted when MXene is used as anode, and Figure 8b shows Na diffusion path for V_2_B_2_ as MBenes.^[^ ^48^ ^]^ The multivalency of boron, its electron‐deficient properties, and its effective redox chemistry with boron and lithium atoms make MBenes highly promising for high energy and high‐power densities. Hence, MBenes are a promising material for advanced battery applications.^[^ ^118^ ^]^ It is noted that theoretical predictions have highlighted Fe_2_B_2_ and Mo_2_B_2_ as promising candidates for use in LIBs.^[^ ^22^ ^]^

In addition, a collection of hexagonal MBenes, which included V_2_B_2_, Y_2_B_2_, Mo_2_B_2_, Cr_2_B_2_, Ti_2_B_2_, Zr_2_B_2_, and Sc_2_B_2,_ is studied.^[^ ^116^ ^]^ Ti_2_B_2_ monolayer was selected as the anode material for SIBs and LIBs based on DFT calculations.^[^ ^116^ ^]^ The study found that the interaction between isolated lithium atoms and MBenes, indicated by a substantial negative adsorption energy, enhances the reversibility and safety of lithium‐ion batteries (LIBs) by preventing metallic lithium formation. The specific capacities of 2D Fe_2_B_2_ and Mo_2_B_2_ as LIB electrodes were ≈444 and 665 mAh g^−1^, respectively, outperforming some other 2D materials. With similar energy barriers for lithium diffusion, MBenes demonstrate exceptional lithium storage capacities and strong potential as anode materials for LIBs.^[^ ^50^ ^]^ An investigation by Zha et al.^[^ ^56^ ^]^ utilized first‐fundamentals computations to predict that H‐ and T‐type Mo_2_B would be promising anode materials for lithium‐ion batteries (LIBs). In contrast to Mo_2_C,^[^ ^130^ ^]^ both T‐ and H‐type Mo_2_B showed similar conductivities but with noticeably greater thermal conductivities. In addition to having a remarkably low 0.037 eV migration barrier, H‐type Mo_2_B has a theoretical volume capacity of 2424 mAh cm^−^^3^. In addition, it is known to be a steady structure that can change into T‐type Mo_2_B under strain.^[^ ^122^ ^]^

**Figure 9:**
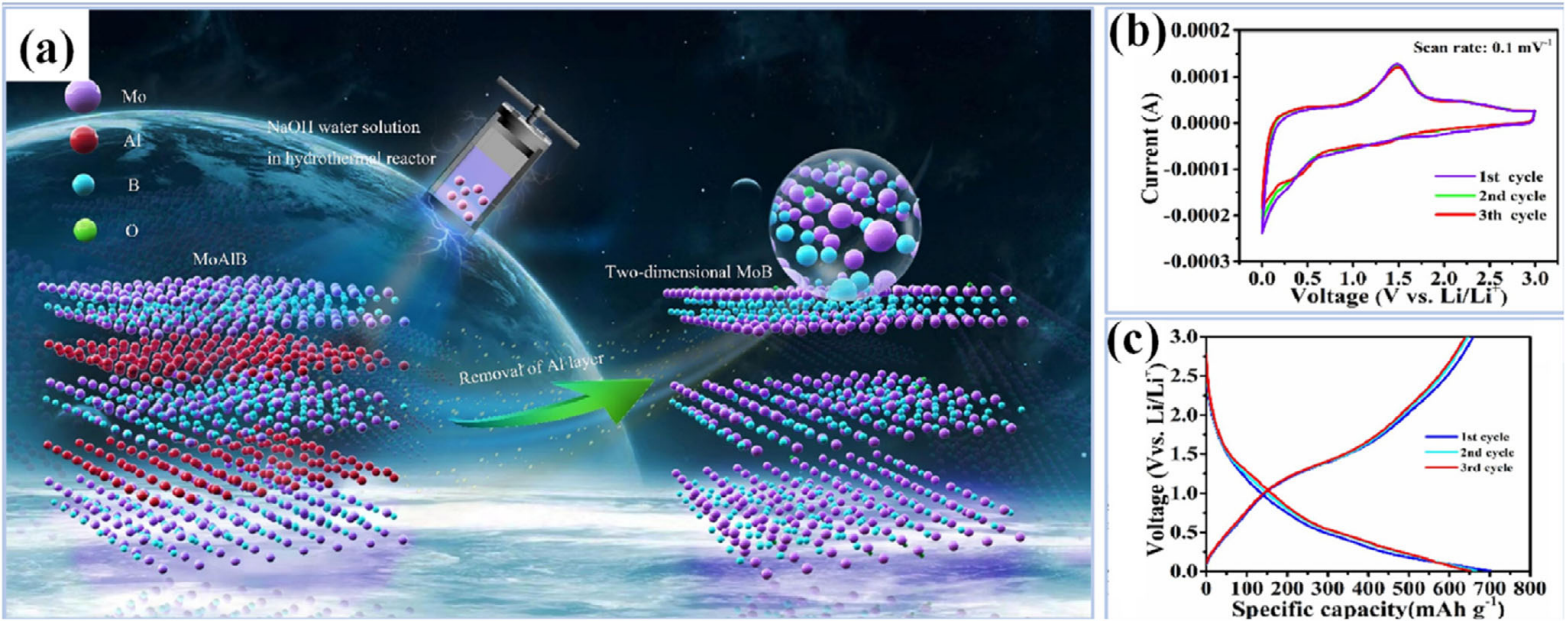
The schematic illustration of a) the formation of 2D MoB MBene, b) cyclic voltammetry (CV) curves at a scan rate of 0.1 mV s^−1^. c) Voltage profiles at 50 mA g^−1^ (Adapted with permission).^[^ ^94^ ^]^

The production of 2D MoB MBene through NaOH etching of MoAlB precursors (**Figure** **9**a) results in a distinctive accordion‐like layered morphology that significantly enhances its electrochemical properties for lithium‐ion batteries. Cyclic voltammetry analysis (1 mV s^−1^ scan rate, Figure 9b) identifies reversible redox activity at 1.20/1.49 V, confirming stable lithium‐ion intercalation. Initial charge/discharge cycles (Figure 9c) achieve a remarkable capacity of 701.7 mAh g^−1^ at 50 mA g^−1^, with Coulombic efficiency stabilizing at 98% by the third cycle. Comparative cycling tests demonstrate MoB's exceptional performance (671.6 mAh g^−1^) versus unetched MoAlB (<50 mAh g^−1^) after 50 cycles, highlighting the benefits of aluminum removal for creating active sites. The strong initial capacity and stable cycling performance are mainly due to the expanded layers and increased surface area after etching, which makes it easier for lithium ions to move and react with the active sites. The slight drop in capacity over time is likely caused by internal stress and resistance building up during repeated charging and discharging. Rate performance evaluation reveals excellent capacity retention (93.8%) at 0.1 A g^−1^, though kinetic constraints become apparent at higher currents (33.1% retention at 5 A g^−1^). Detailed analysis shows progressive capacity retention of 88.6% (0.2 A g^−1^), 79.1% (0.5 A g^−1^), 73.1% (1.0 A g^−1^), 62.1% (2.0 A g^−1^), and 33.1% (5.0 A g^−1^) relative to the 0.05 A g^−1^ baseline. Extended cycling tests confirm stable operation with 144.2 mAh g^−1^ remaining after 1000 cycles.

Comparative studies of Mo_2_AlB_2_ demonstrate significant performance improvements through mechanical milling. Post‐etching CV profiles reveal new redox activity at 0.39/0.45 and 1.25/1.52 V versus Li/Li⁺. The *b*‐value analysis (0.70–0.82) suggests a combined diffusion‐controlled and capacitive storage mechanism. Milled samples exhibit fourfold capacity enhancement and maintain 302 mAh g^−1^ at 200 mA g^−1^. Due to milling, the size of MBene flakes gets reduced, which is beneficial for shortening the diffusion pathway and thus, increases ionic conductivity. In addition, reduced size opens more active sites for Li^+^ storage. The increase in discharge capacity after 200th cycle indicates formation of new storage sites and enhancement of ionic diffusivity with repeated cycling. It is noted that exfoliation for longer times further facilitates this phenomenon. These experimental results, supported by DFT calculations showing favorable Li⁺ diffusion barriers (0.30 eV),^[^ ^131^ ^]^ establish MBenes as superior anode materials compared to conventional graphite (372 mAh g^−1^) and MXenes (320 mAh g^−1^).^[^ ^123^ ^]^

**Figure 10:**
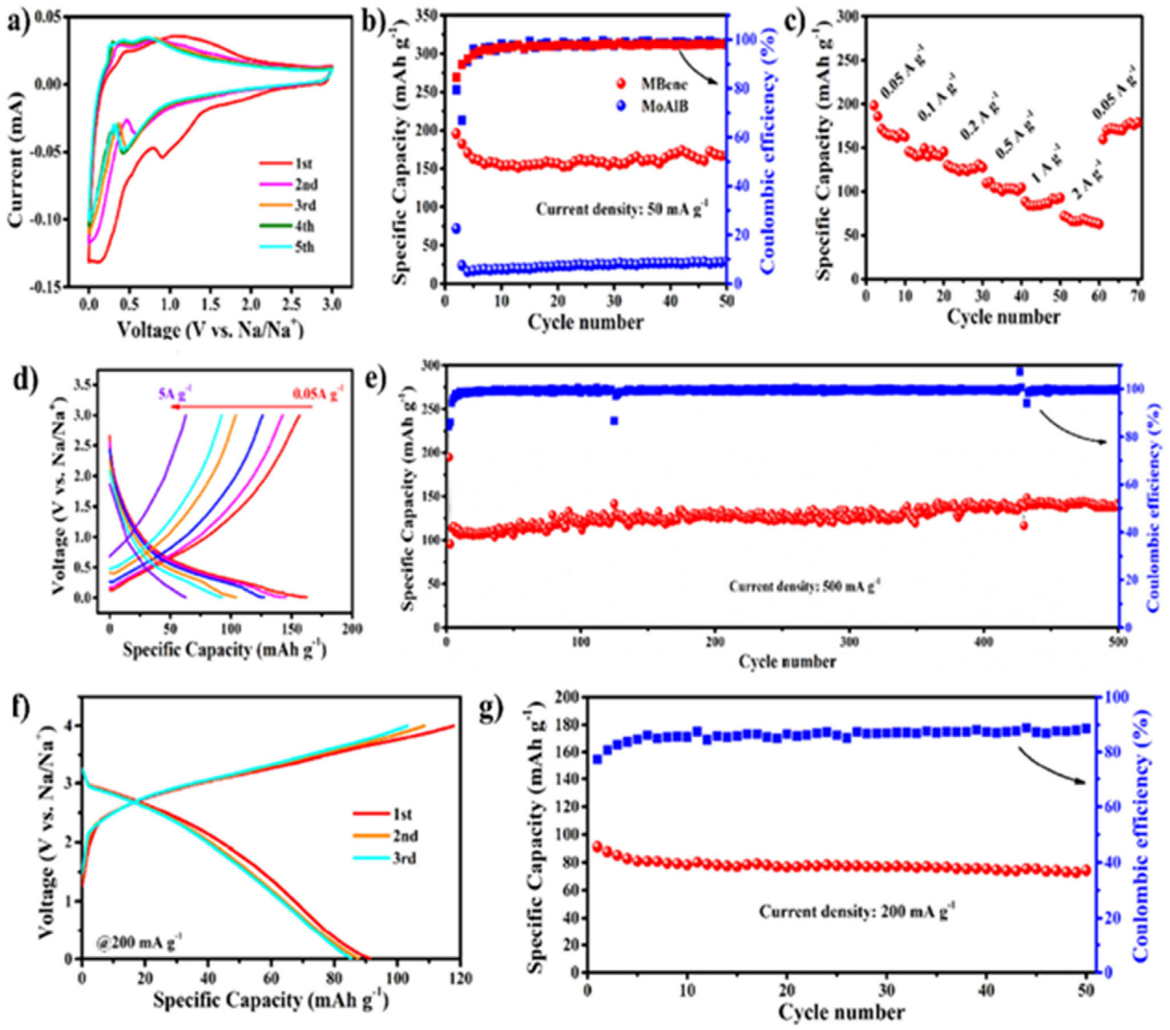
Electrochemical performance of MoB MBene anodes in SIBs. a) CV curves recorded at a scan rate of 0.1 mV s^−1^. b) Cycling performance comparison between MoAlB and MoB MBenes at 50 mA g^−1^. c) Specific capacities of MoB MBenes at various current densities, and d) corresponding voltage profiles. e) Cycling stability and Coulombic efficiency of MoB MBenes at 500 mA g^−1^. Electrochemical performance of a MoB/NVP full cell: f) charge/discharge curves and g) cycling performance at a current density of 200 mA g^−1^. (Adapted with permission).^[^ ^71^ ^]^

The unique accordion‐like structure of 2D MoB MBene demonstrates superior sodium‐ion storage capabilities (**Figure** **10**). Initial cyclic voltammetry analysis (0.1 mV s^−1^ scan rate, Figure 10a) reveals redox activity at 0.91/1.05 V, shifting to 0.43–0.60 V after solid‐electrolyte interface formation, with consistent overlapping curves from the third cycle confirming electrochemical stability.^[^ ^132^ ^]^ Galvanostatic measurements at 50 mA g^−1^ show initial discharge/charge capacities of 165.3/195.7 mAh g^−1^, stabilizing at 153–163 mAhg^−1^ with 94% Coulombic efficiency—significantly outperforming conventional carbon anodes.^[^ ^133^ ^]^

Comparative cycling tests (Figure 10b) highlight MoB's enhanced performance (166.4 mAh g^−1^ after 50 cycles) versus unmodified MoAlB (≈25 mAh g^−1^), demonstrating the critical role of aluminum removal in creating efficient sodium‐ion transport pathways. The material exhibits excellent rate capability (Figure 10c) with capacities ranging from 162.9 mAh g^−1^ (0.05 A g^−1^) to 62.2 mAh g^−1^ (2.0 A g^−1^), corresponding to retention rates of 88.8%–38.2% (Figure 10d). Extended cycling at 500 mA g^−1^ (Figure 10e) maintains 138.6 mAh g^−1^ after 500 cycles, surpassing the performance of comparable MXene materials. Complete cell configurations using Na_3_V_2_(PO_4_)_3_ cathodes (Figure 10f,g) deliver initial capacities of 91.3 mAh g^−1^, retaining 74.3 mAh g^−1^ (81.4% capacity retention) after 50 cycles. Electrochemical impedance spectroscopy confirms excellent charge transfer characteristics (≈20 Ω resistance) and stable sodium‐ion diffusion kinetics. Post‐cycling structural analysis verifies the material's exceptional morphological stability throughout extended electrochemical operation.^[^ ^71^ ^]^

**Figure 11:**
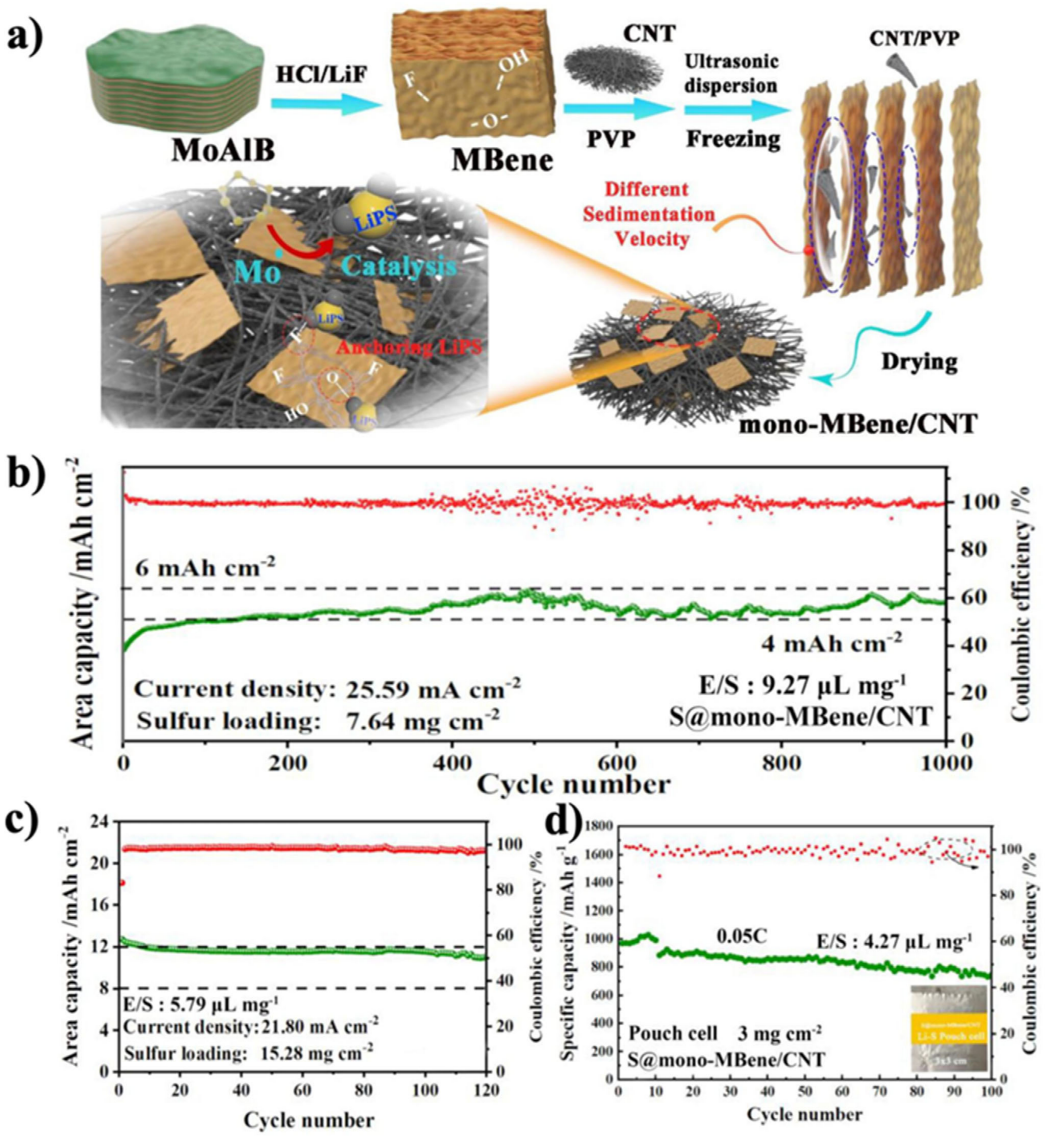
The outcomes of S@MoB/CNT as a Li–S battery cathode. a) Diagram of the ice template used to prepare MoB/CNT. b) The long‐term cycling performance of high sulfur loading cells based on S@mono‐MBene/CNT cathode. c) Ultra‐high sulfur loading cells with S@mono‐MBene/CNT cathode cycling performance. d) Cycling efficiency of a pouch cell at 0.05 using a S@mono‐MBene/CNT cathode. (Adapted with permission).^[^ ^134^ ^]^

As a result of recent developments in battery technology, a composite material called MoB/CNT was created using the ice template method. This material has potential as a cathode host for lithium–sulfur batteries. Using the ice template method, a composite of monolayer MoB and carbon nanotubes (CNT) has been developed as a lithium–sulfur battery cathode host (**Figure** **11**a). The exposed locations of MoB nanosheets act as agents to anchor lithium polysulfides and transform them into Li_2_S_2_/Li_2_S. This results in an elevated sulfur loading of more than 15 mg cm^−^^2^ and steady cyclability for more than 1000 cycles at a rate of 4 mAh/cm^2^ (Figure 11b–d).^[^ ^134^ ^]^ Functionalization is highlighted as a promising research avenue in this section, which also encapsulates the electrochemical energy storage performance of pristine MBenes. For instance, metal atoms adsorb more readily on oxygen‐functionalized V_2_B_2_, however, this causes the surface diffusion energy barrier to fluctuate between rising and falling.^[^ ^119^ ^]^

Excellent conductivity and fully exposed surface sites are essential for catalysts used in a variety of reactions, such as carbon dioxide reduction (CO_2_RR), nitric oxide reduction (NORR), nitrogen reduction (NRR), the evolution of hydrogen (HER), oxygen reduction (ORR), oxygen evolution (OER), and nitrate reduction (NO_3_RR).^[^ ^135^ ^]^

In addition, Zhang et al. utilized FeB_2_ as a structure to examine the nitrogen reduction reaction (NRR), and they found that MBenes work via a tandem electrocatalytic process. The B site produces intermediates by activating NO_3_^−^, according to charge studies, whereas the Fe site separates water molecules and supplies hydrogen to the B site. Through this process, FeB_2_ was able to produce NH_3_ at a pace of 25.5 mg h^−1^ cm^2^ with a Faraday efficiency of 96.8%.^[^ ^136^ ^]^ Urea can be produced directly by the catalytic reactions of carbon dioxide and nitrogen or by their combined participation in a single catalytic pathway. Mo_2_B_2_ and Cr_2_B_2_ have superior overpotentials of −0.94 and −0.52 V, correspondingly, when compared to Pd–Cu catalysts, according to Zhu et al. Because the nitrogen reduction reaction (NRR) catalytic site is wrapped by ‐OH groups in aqueous conditions, this catalytic reaction exhibits useful selectivity.^[^ ^137^ ^]^ Since greenhouse gases such as carbon dioxide have the ability to absorb long‐wave radiation from the ground, they can produce the greenhouse effect and raise surface temperatures.^[^ ^138^ ^]^ Liu et al. typically investigated the catalytic ability of certain M_2_B_2_‐MBenes (M = Cr, Fe, Mn, and Co) to decrease carbon dioxide, and it was revealed that Mo_2_B_2_ and Cr_2_B_2_ have excellent catalytic activity and selectivity for the reduction of carbon dioxide to methane.^[^ ^139^ ^]^ Different reaction pathways can be produced by different kinds of MBenes. For instance, when Xiao et al. examined the catalytic activity of certain M_3_B_4_ and M_2_B_2_ type MBenes for CO_2_ reduction, they discovered that V_3_B_4_ was selective in its ability to convert carbon dioxide into either methanol or methane.^[^ ^140^ ^]^

In addition to being a crucial component of chemicals and fertilizers, ammonia (NH_3_) is necessary for daily living.^[^ ^141^ ^]^ Electrocatalytic nitrogen reduction reaction (NRR) offers a promising alternative to the Haber–Bosch process due to its energy efficiency, operational flexibility, and simpler reaction conditions.^[^ ^142^ ^]^ In addition, the present electrolytic method is hindered by significant overpotential and inadequate reaction kinetics due to the sluggish activation of chemically inert N–N triple bonds.^[^ ^143^ ^]^ In the electrocatalytic denitrification process, the HER mechanism is known to compete with NRR.^[^ ^144^ ^]^ Minimizing the HER reactions that take place at the catalyst surface is crucial when choosing a catalyst.^[^ ^145^ ^]^ Finding an electrocatalytic NRR system that accomplishes low overpotential and high Faraday effectiveness (FE) has proven to be a difficult undertaking.^[^ ^146^ ^]^ Li et al.^[^ ^147^ ^]^ demonstrated the electrocatalytic efficiency of six MB (M = Sc, V, Mo, Cr, Ti, and W) single layers for NRR using basic principles calculations. In contrast to VB monolayers in the final faced setup, analyses showed that N_2_ molecules may be efficiently formed on the surface of MB monolayers, which can initiate the NRR process. According to the data, single layers of VB, CrB, and MoB have exceptional catalytic efficiency for the nitrogen reduction reaction (NRR) and are probably going to be useful catalysts. Using DFT simulations, Wang et al. employed a comparable methodology to study Mo_2_B_2_’s function as a catalyst for NRR.^[^ ^148^ ^]^

It is noted that according to metal/B atom ratios, MBenes are divided into three categories:

FeB_2_, RuB_2_, and OsB_2_ (3.70–3.93 Å) belong to group 1, which has a collapsed metal layer with a B atom layer on the surface. V_3_B_4_, Nb_3_B_4_, and Ta_3_B_4_ (4.84–5.26 Å) have two B layers sandwiched between three metal layers in group 2. The metal‐covered surface of CrB, MnB, ZrB, and HfB (2.23–2.85 Å) is a bilayer structure in group3.^[^ ^149^ ^]^ It is noted that the metal atoms in Groups 2 and 3 (metal active surface) and the accessible B atoms in Group 1 (B active surface) are dynamic sites for N_2_ absorption. Bonding orders of 0.26 to 1.31 and M–B bonding lengths of 1.91 to 2.43 Å are observed in MBenes.^[^ ^149^ ^]^ According to the localized electron functions, these values show a strong covalent link between the B and metal atoms.^[^ ^150^ ^]^ However, the anti‐bonding π* orbital of N_2_ can receive electrons from the occupied orbitals of MBenes, which makes bonding with N_2_ easier. The functional roles of 2D MXenes, which range from 5.2 to 6.6 eV, are significantly higher than those of MBenes, which range from 3.38 to 5.05 eV.^[^ ^151^ ^]^ Metal catalysts must effectively absorb N_2_ for the nitrogen reduction reaction (NRR) to occur. However, B and metal‐activated surfaces, accordingly, and the end‐on and side‐by‐side arrangements in which N_2_ molecules can chemisorb onto MBenes. While both N atoms form bonds with nearby B or metal atoms in the side‐by‐side configuration, one N atom attaches to the activation site in the end‐on configuration.^[^ ^149^ ^]^ Using three techniques, researchers evaluated the possible viability of nitrogen reduction reaction (NRR) for all MBene units. The most effective method was the enzymatic one, which produced the lowest NRR probability at both the metal‐activated and B surfaces. It is reported that initial potentials (U) throughout MBene frameworks. B functional surfaces display U values between 0.03 and 0.26 V, whereas metal functional surfaces display U values between 0.16 and 0.92 V. A density of states (DOS) analysis for MBenes could provide more information, where all MBenes exhibit metallic characteristics and measurable Fermi levels.^[^ ^149^ ^]^ The density of states (DOS) at the Fermi levels is strongly influenced by the presence of B atoms on the energetic surfaces.^[^ ^149^ ^]^ All things considered, these results demonstrate the MBenes' promising potential as efficient catalysts for the nitrogen reduction reaction, highlighting the importance of their structural traits and electrical characteristics in promoting N_2_ reactivity and absorption. Lin et al.^[^ ^152^ ^]^ investigated Cr_2_B_2_’s catalytic properties for NRR. Using N_2_ adsorption on Cr–B bonding, B–B bonding, and the placement of Cr and B atoms in their ideal positions, this study assessed four configurations. The findings showed that the N_2_ molecules had an optimal adsorption energy of 1.235 eV and were successfully pushed to the neighboring Cr‐B bond. The adsorption method required at a least current of 0.29 V and demonstrated remarkable catalytic activity. Xiao et al. discovered that Ta_3_B_4_, Nb_3_B_4_, CrMnB_2_, Mo_2_B_2_, Ti_2_B_2_, and W_2_B_2_ have the lowest maximum potential and current for generating N2 chemical protonation, indicating superior stability and usefulness in electrolytic nitrogen reduction reaction (NRR).^[^ ^64^ ^]^ Furthermore, B_2_@Mo_2_B_2_O_2_ exhibits outstanding stability because of its lower bonding potential of −1.72 eV with NH_3_ as opposed to −2.14 eV with N_2_, which makes it easy to release the NH_3_ molecule.^[^ ^149^ ^]^

In summary, MBenes offer a valuable opportunity for enhancing energy storage and catalytic functions, especially in lithium‐ion and sodium‐ion batteries, thanks to their impressive theoretical capacities and advantageous diffusion properties. Their distinct structural characteristics improve their efficacy as catalysts for nitrogen and carbon dioxide reduction, positioning them as viable alternatives to traditional materials such as MXenes. The ongoing investigation into their functionalization and interaction with metal ions is expected to reveal additional potential in diverse electrochemical processes, reinforcing their significance in upcoming energy technologies. Furthermore, the remarkable stability and efficiency exhibited by MBenes in catalytic reactions highlight their potential for sustainable energy solutions. Future investigations aimed at enhancing their structural and electronic characteristics will be essential in realizing their complete capabilities for conversion applications and energy storage.

## 6 Challenges Related to the Stability of MBenes

MBenes, a kind of 2D transition metal borides, face several stability issues that hinder their synthesis and practical applications. Producing high‐quality MBene nanosheets is challenging due to the complexity of methods like top‐down exfoliation and chemical vapor deposition.^[^ ^117^ ^]^ In addition, surface defects that occur during the synthesis process can reduce the material's stability and affect its overall properties.^[^ ^153^ ^]^ The control of defect chemistry is also critical, as it directly influences MBenes' electronic and catalytic performance, requiring careful management during synthesis.^[^ ^154^ ^]^ Structural challenges, such as those encountered during molecular beam epitaxy (MBE) growth, can result in marginally stable structures, with factors like Schwoebel barriers contributing to instability over time. Another major concern is the rapid oxidation of MBene layers, underscoring the need for effective passivation strategies to preserve their stability.^[^ ^153^ ^]^ When used in applications like perovskite solar cells, MBenes can improve charge transfer but may also introduce interface defects, which can undermine long‐term performance.^[^ ^155^ ^]^ Moreover, their sensitivity to environmental conditions poses a significant obstacle for practical use, as variations in the environment can degrade their performance.^[^ ^154^ ^]^ Overcoming these stability issues is essential to fully leveraging the potential of MBenes in advanced technologies.

While the techniques discussed significantly enhance the stability of MBenes, challenges remain in scaling these methods for industrial applications. Further research is needed to optimize these processes and fully realize the potential of MBenes in various technological domains. Surface engineering and composite material design are essential for improving the stability and functionality of MBenes, especially in perovskite solar cells (PSCs) and energy storage systems. These approaches enhance charge transfer, reduce defects, and fine‐tune the material properties of MBenes, boosting efficiency and durability. For example, interface bridging techniques in PSCs facilitate electron transport by introducing additional charge carriers and repairing surface imperfections.^[^ ^155^ ^]^ The creation of interfacial dipole moments also improves electron collection, increasing overall device efficiency.^[^ ^155^ ^]^ Additionally, MBenes helps optimize perovskite crystal formation, leading to higher‐quality films with fewer defects—critical for long‐term stability.^[^ ^155^ ^]^

Another effective method is aluminum etching, where controlled removal of aluminum from MoAlB produces MBene‐MoB, strengthening structural stability and conductivity in electrochemical applications. Such advancements have already pushed PSC efficiencies to 24.32% while maintaining operational stability,^[^ ^155^ ^]^ with studies showing sustained performance over extended periods. Composite material design further enhances MBene stability. Methods like liquid‐phase exfoliation yield high‐purity MBene nanosheets, while chemical vapor deposition (CVD) ensures uniform growth on substrates, improving durability. Certain MBenes, such as ScB and TiB, demonstrate low energy barriers for lithium and sodium ion diffusion, positioning them as ideal anode materials for high‐performance batteries.^[^ ^127^ ^]^ These composites also promote better crystal growth and charge mobility, expanding MBenes' potential in energy technology. Despite these advantages, challenges persist in scaling these techniques for industrial use, requiring further research to refine their application across different device designs.

The practical application of MBenes has been limited due to the challenging and intricate synthesis methods, largely because of the difficulty in accurately distinguishing tertiary orthorhombic MAB (ort‐MAB) phases.^[^ ^102^ ^]^ Research has concentrated on the tertiary ort‐MAB phases, such as MoAlB and Cr_2_AlB_2_, to produce CrB and MoB ort‐MBenes. However, these MoB and CrB ort‐MBenes demonstrated low performance, often resulting from excessive etching of Al or complete dissolution of the primary phases.^[^ ^47^, ^156^, ^157^ ^]^ Developing ort‐MBenes faces two main obstacles: first, there are no reliable, consistent, clean ort‐MAB phases available for etching tests, and second, the difficulty in etching these phases is due to issues like oxidation, dissolution, and potential recrystallization when Al layers are removed from tertiary ort‐MAB phases.^[^ ^158^ ^]^ Researchers have also identified the first hexagonal tertiary MAB (h‐MAB) phase, Ti_2_InB_2_, and created the multilayer boride TiB by etching away in layers. However, TiB underwent significant phase transitions at high temperatures.^[^ ^49^ ^]^ Rosen et al. recently developed two hexagonal quaternary MAB phases, called i‐MABs, namely (Mo_2/3_Y_1/3_)_2_AlB_2_ and (Mo_2/3_Sc_1/3_)_2_AlB_2_. They selectively removed the Al and Y (or Al and Sc) atoms to create a new 2D structure, referred to as “boridene” or i‐MBene,^[^ ^36^, ^95^, ^158^, ^159^, ^160^, ^161^, ^162^, ^163^ ^]^ marking a major step forward in MAB exfoliation research. Wang et al.^[^ ^132^ ^]^ combined extensive computational screenings with precise calculations to identify 30 thermally stable and 103 metastable h‐MAB phases. These phases belong to models with stoichiometries of M_2_AB (model 211), M_2_AB_2_ (model 212), and M_3_AB_4_ (model 314).^[^ ^51^ ^]^ Among all these systems, 81 are predicted to undergo successful etching to form 20 h‐MBenes, with tertiary h‐MAB phases proving to be better precursors for 2D MBenes compared to earlier tertiary ort‐MAB phases.

A total of 140 M‐A‐B systems were analyzed by researchers. Among all these systems, 139 exhibited thermally stable MAB phases, following compositions of M_2_AB_2_ (212), M_3_AB_4_ (314), and M_2_AB (211). The team also calculated the enthalpies (δH) for the stability or metastability of these MAB phases, with a specific focus on three‐phase mixtures. Among the 133 predicted h‐MAB phases, 30 were found to be thermally stable, while the remaining 103 were metastable, with a δH value around 70 meV/atom. Model 212 had the most stable (13) and metastable (42) phases. These structures exhibited space groups either in hexagonal P6m2 (No. 187) or P63/mmc (No. 194).^[^ ^149^ ^]^ As for the predicted hexagonal 314 structure, its space group remained consistent with that of the 212 h‐MAB (P6m2 No. 187). However, the M‐B layers in the 314 model were thicker compared to those in 212, with five layers of M and B compared to the three in h‐M_2_AB_2_.^[^ ^149^ ^]^

## 7 Conclusion

The potential of 2D transition metal borides (MBenes) is thoroughly examined in this review, emphasizing their potential contribution to the development of energy storage systems. Superior electrochemical characteristics, such as strong conductivity, a greater capacity for energy storage, and exceptional structural stability, make MBenes a desirable substitute for existing 2D materials such as MXenes. Because of their strong mechanical properties, high theoretical capacity, and low diffusion energy barriers, all of which are essential for enhancing cycling stability and performance, they perform well, especially when used as anode materials for lithium‐ion batteries (LIBs). Beyond energy storage, functionalized MBenes have a wide range of uses in fields including lithium–sulfur batteries and nitrogen reduction catalysis, where their special qualities offer significant benefits over traditional materials. The adaptability of MBenes is increased by the capacity to customize their surface capabilities, which makes them appropriate for a wide range of energy‐related technologies.

Notwithstanding these outstanding qualities, several obstacles still stand in the way of MBenes' potential being fully realized. Their stability and performance in real‐world applications are greatly impacted by severe restrictions like as surface oxidation and flaws. When thinking about the scalability of MBenes for large‐scale energy storage devices, these concerns are very relevant. Therefore, the creation of environmentally friendly and scalable synthesis techniques is crucial to overcoming these obstacles. However, methods such as chemical and hydrothermal exfoliation show promise; more refinement is needed to guarantee cost‐effectiveness, uniformity, and reproducibility in commercial settings. Furthermore, although MBenes are superior to MXenes in some applications, research is still ongoing to determine their long‐term stability and practical performance. The durability of MBenes requires further investigation, particularly in settings where oxidation or other types of deterioration are likely to occur. In addition, more research is needed to fully understand the range of their uses in next‐generation energy systems, such as supercapacitors and hydrogen storage, among others.

To sum up, even though MBenes are a very promising class of materials that could transform energy storage and catalysis, more experimental study is needed to improve their stability, streamline their synthesis, and maximize their incorporation into commercial energy technologies. To fully realize the potential of MBenes and secure their place in the future of energy technology, these problems must be resolved in addition to the creation of more scalable and sustainable synthesis methods. To fulfill the increasing need for clean energy solutions worldwide, more effective, sustainable, and ecologically friendly energy storage technologies may eventually be developed in large part due to MBenes' ongoing development.

## Conflict of Interest

The authors declare no conflict of interest.

## Author Contributions

T.A. and S.H. wrote the original draft and contributed to data curation. M.B. and A.R. reviewed & edited the final manuscript. A.R. acquired funding.
